# Birth Defects in Gaza: Prevalence, Types, Familiarity and Correlation with Environmental Factors

**DOI:** 10.3390/ijerph9051732

**Published:** 2012-05-07

**Authors:** Awny Naim, Hedaya Al Dalies, Mohammed El Balawi, Eman Salem, Kholud Al Meziny, Raneem Al Shawwa, Roberto Minutolo, Paola Manduca

**Affiliations:** 1 Palestinian Energy Authority, Gaza, Palestine; Email: awnynaim@gmail.com; 2 Al Shifa Hospital, Gaza, Palestine; Email: dr_hyhd@hotmail.com (H.A.D.); dr_mgb10@hotmail.com (M.E.B.); memisalem@yahoo.fr (E.S.); lolomnm@hotmail.com (K.A.M.); 3 IT consultant, Gaza, Palestine; Email: never.say.n0.way@hotmail.com; 4 Nephrology Department, University of Naples, Via Giovanni Paladino, 80138 Napoli, Italy; Email: roberto.minutolo@unina2.it; 5 Genetics Department, University of Genoa, C.Europa 26, 16132 Genova, Italy

**Keywords:** registration at birth, major structural birth defects, familiarity of birth defects, exposure to war related events, environmental teratogens

## Abstract

This is the first report of registration at birth, and of incidence of major structural birth defects (BD) obtained in Gaza at Al Shifa Hospital, where 28% of total births in Gaza Strip occur. Doctors registered 4,027 deliveries, with a protocol comprehensive of clinical, demographic, kin and environmental questions. Prevalence of BD is 14/1,000, without association with intermarriage or gender of the child. Prevalence of late miscarriages and still births are respectively 23.3/1,000 and 7.4/1,000, and of premature births 19.6/1,000. Couples with a BD child have about 10 times higher frequency of recurrence of a BD in their progeny than those with normal children, but none of their 694 siblings and only 10/1,000 of their 1,423 progeny had BD, similar to the frequency in general population. These data suggest occurrence of novel genetic and epigenetic events in determination of BD. Children with BD were born with higher frequency (*p* < 0 001) in families where one or both parents were under “white phosphorus” attack, that in the general population. Bombing of the family home and removal of the rubble were also frequently reported by couples with BD occurrence. These data suggests a causative/favoring role of acute exposure of parents to the weapons-associated contaminants, and/or of their chronic exposure from their persistence in the environment on the embryonic development of their children.

## 1. Introduction

Our main aim was to describe reproductive health with respect to prevalence of major structural congenital anomalies (BD), late miscarriages (M) and still (SB) and premature (P) births in delivery at Al Shifa Hospital, the major maternity hospital of Gaza [1,2], and to assess the utility of the comprehensive protocol for registration utilized, inclusive of environmental questions related to exposure to war events. The resulting data will be the baseline for a continuing implementation of the registry, as the primary mean to investigate the quality of the reproductive health and to monitor changes in time in the general health of the population [3].

The collection of the data on prevalence of birth defects was done according to the local specificities and with modalities that allow also interpretation of changes over time and comparison with other registers from around the world.

BD incidence reflects allelic frequency of genes in the population and varies with its intermarriage rate, but is also susceptible to environmental changes [4,5] and has been linked to various sources of pollution, nourishment, mother health, infectious diseases and acute environmental changes of various nature, including use of modern metal augmented weaponry that have teratogen and/or mutagen effects [6,7,8,9]. Information on the health of siblings of parents and their kin contributes to reinforce, or undermines, the relevance of direct and deterministic genetic contribution for the specific BD in a family. Information on environmental events provides clues of their potential relevance [10]. Within each class of defect, there is phenotypic variation and some phenotypes are associated with known genetic mutation(s) while for others, the majority still, there is no genetic determinant identified. The change in gene’s functionality can be consequence of mutation or be an epigenetic change or a resultant of both. In many cases maternal environment (including genetic background and external environmental effectors) is involved in the determination of the expression of the phenotype of BD [5,8,9]. In particular, for some of most frequently found BD, is known that the molecular requirements for neural tube closure are complex and implicate a variety of genes, pathways and cellular functions and congenital neural tube defects (NTD) have multifactorial etiology [11]. Congenital polycystic kidney disease (PKD) is usually associated to homozygosis for mutations in the PKHD1 gene, with variable penetrance [12]. High prevalence of PKD is recorded in Arab countries, favored by prevalent high rates of intermarriage [13]. For about 30% of CHD and Tetralogy of Fallot (TOF) there is association with wide genomic rearrangements, and cohort studies have detected genetic mutations in single genes putatively involved in CHD, with very low frequency [14]. Expression of the CHD is susceptible to environmental effectors. Inheritance of cleft lip and palate in mice is due to combinations of genetic and not genetic factors [15]. Synpolydactyly is linked to mutations in the gene Hoxd13, but manifestation of the phenotype is dependent upon environmental factors [16].

Presence of teratogen elements in the post-war environment is expected in Gaza after Operation Cast Lead, because of the kind of metals detected in the weapon systems used [17,18]. These have low mutagen but high teratogen and carcinogen potentials, cannot be eliminated from the environment and are capable to act as metalloestrogens affecting multiple cellular pathways during embryo and fetal development [19,20,21]. None of the pregnancies here registered occurred during the major war events when the WP and bombs were used (*i.e*., in the Cast Lead operation). Metal load in living organisms is cumulative, and its effects, if any, show over time. We here investigated if there are long term effects on reproductive health that can be associated to cumulative, chronic exposures due to persistence in the environment of not removed toxicants, like the above mentioned endocrine disruptors and metals derived from weapons.

The traditional hyphotesis behind prevalence data surveillance is that detection of a particularly high prevalence of BD in one particular population, or a sudden increase in prevalence over time, points to the existence of some novel environmental cause [22,23]. From war theaters where similar weapons were used, the time for teratogen effects to manifest themselves may be on the order of 2–4 years [6]. These circumstances, in lack of previous record of births and data bank of BD, make relevant to have begun now the recording the prevalence of BD and the state of reproductive health in Gaza.

Our protocol was devised in order to collect demographic and health data, and kin information on the couples with present reproductive problems (BD, M. SB and P) and those with previous events of BD (PBD). For all these, and for normal newborn, parent’s environmental exposure to White Phosphorus (WP), and for BD children parent’s also to bombing, were registered. The questions about environmental exposure were chosen, as occurs in each country, to include also information about the specific circumstances of the country, including environmental factors induced by military attacks.

## 2. Results and Discussion

### 2.1. Registration of Births

We report registration, documentation of kin and environmental exposure for 55 BD, 94 late miscarriages, and 30 still born deliveries in a five month period among the 4,027 interviews conducted at birth (Table 1 In addition, 47 prematurely born were separately registered only in the last three months.

The prevalence of major structural birth defects by clinical diagnosis in Al Shifa is 14.0/1,000 of live births. Prevalence of late miscarriages, premature and still born children and the frequency of women who delivered a normal child but had a previous child with birth defect are also listed in Table 2. The sex ratio of normal newborns is 1 and, while there is no obvious prevalence of sex in BD and PBD and in M, prematures and SB are preferentially females.

The intermarriage rate among first cousins is high in Gaza, nonetheless is not different in couples with BD child or SB than in the population as whole. Significant higher rate of intermarriage are seen for M, and in the couples that had BD in past times (PBD). Premature births occur most frequently in couples where the parents are unrelated (Table 2).

**Table 1:** Summary of the question asked during registration of the births in delivery room. * confirmation of the diagnosis was received by the Doctors in the Intensive Care Unit. (**A**) Section for all deliveries; (**B**) Section for deliveries with BD, premature and still births, and late miscarriages.

(**A**) For all deliveries	(**B**) For parents of BD children, P, PBD, and late M
Name of parents Hospital ID	Anamnesis of mother and father
Family residence	Number of brothers and sisters of mother and father and their health status
Child sex	Number of children of brothers and sister of mother and father and their health status
Children date of birth	Type of dwelling
Weeks at delivery	Water access
Modality of delivery	Bombing on residence, in presence or when absent
Child weight	Bombing in adjacent housing
Child health conditions	Attacks to residence by white phosphorus, in presence or when absent
Clinical observations	Wounds, burns, injuries reported, which injuries
Child diagnosis	Care of other family members who received injury and which kind of injury
Congenital major structural birth defect from a list of major ones *	Clean up bombed, attacked residence
Consent of parents to use professionally the information	Use of recovery materials from debris to rebuild housing
Occupation of parents	Personally worked or familiar at home worked in recycling materials form rubble
List of other children of the couple and note on their health status: if alive, dead (and cause of death), previous miscarriages (numbers, time, the week they occurred and if the fetus had BD)	Children of the family play in bombed/attacked grounds
Direct exposure to WP and WP attacks on their residence in their absence	Health problems during attacks 2006 and 2008/09, description
Information if cases of BD occurred among the neighbors	

### 2.2. Classification of BD Types and Family History

Past occurrence of BD in the reproductive history of all couples, has a frequency of 2.09% and they include BD occurring between 1 and 18 years ago. The data for PBD have no value as incidence or prevalence data, and were only collected to analyze the recurrence of BD in a couple. s-Tables II to s-Table IV show the data in full.

**Table 2:** Prevalence of reproductive problems, sex ratio of newborns and parent’s intermarriage.

New born	Normal	Birth defect	Miscarriage	Premature	Still Born	Previous BD
Total numbers	3,811	55	94	77 *	30	82
*prevalence/1,000*	*974.6*	*14.0*	*23.3*	*19.6*	*7.4*	*20.9 ^a^*
Sex ratio M/F	1 ^b^	0.94	1.1	0.67	0.88	0.95
% Parents first cousins	27 ^b^	29	36,1	13,3	30	39
*P vs*.* Normal*		*0.847*	*0.06*	*0.009*	*0.871*	*0.022*

Prevalence/1,000 for normal children, birth defects and premature births is calculated on live births (3,919), that for late miscarriages and still born is on total deliveries (4,027). ^a^ Frequency/1,000 of previous birth defects (BD) is on live births. All numbers refer to the births registered over a five month period, with the exception of premature, where ^*^ is the value estimated from the actual numbers of premature registered over 3 months, while previously these were registered, according if normal or BD, in the respective lists; ****^b^ The value of reference for sex ratio and parent first cousins couples with a normal newborn are calculated on 200 registered files, chosen randomly out of the total. p refers to the significance of the difference in familiarity of parents, compared to the parents of normal children.

**Figure 1:**
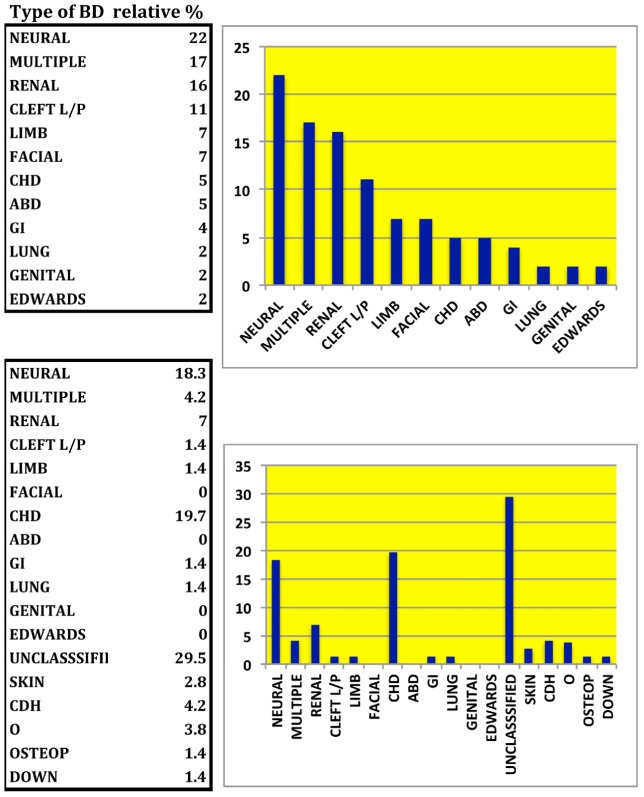
Type of Birth defects. The relative presence among BD (top panel) and PBD (bottom panel) is expressed in percentage of the type of defect: GI gastrointestinal, CHD congenital heart disease, ABD Abodminal wall defect, CDH Congenital displasia of hip, O Others undescribed, OSTEP osteopetrosis.

The types of BD registered are listed in Table 3 and their percentage distribution in Figure 1, together with the distribution of the types of PBD registered among all couples. PBDs includes a relatively high presence of CHD, present at much lower frequency instead in our registration of BD, due to lack of instrumental diagnosis that leaves undetected minor CHD, to the fact that often CHD manifest clinically only in days, weeks or even months after birth, and longer term survival of CHD carriers than other serious BD. Among the BD admissions to the Intensive care Unit about 50% children died within 2 weeks in the year 2010. Death of premature children was about 60% in 2010 [24].

**Table 3:** List of the BD registered at birth. BD classification-Eurocat/ICD10.

	Q0 Ancephaly + multiple malformation
Q20 CHD	Q74. Q74.2 congenital malformations of lower limbs
Q02 Microcephal + IUGR	Q39.0 aresia of oesophagus without fistula
Q75.9 Congenital malformation of skull and face bones + distended abdomen	Q39.0 aresia of oesophagus without fistula, premature
Q41.2 Small bowel volvulus + Menconium cyst	Q0.00 Anencephaly
Q87.0 CL, abnormal ear & mouth shape, deformity in toes	Q05 Spina bifida
Q89.7 and vaginal anomalies, abdominal distension, hydrops fetalis	Q30.0 Choanal atresia
Q98.7 multiple	Q03 hydrocephalus
Q79.5 Congenital malformation abdomen + lung hyperplasia	Q75.9 Facial bones anomaly
Q74.9 Unspecified congenital malformation of limbs	Q37 Ccenter palate with ccenter lip + multiple
Q60.6 Potter syndrome and renal agenesis, premature	Q25 Transposition of the great arteries
Q03 Hydrocephalus	Q62 Hydronephrosis
Q01.2 Occipital encephalocele	Q75.9 Facial bones anomaly
Q60.6 Potter’s syndrome + hypoplastic lungs	Q37 Ccenter palate with ccenter lip
Q75.9 Congenital malformation of skull and face bones, +ascite+IUFD	Q37 Ccenter palate with ccenter lip, premature
Q61.3 Polycystic kidney, unspecified	Q98.7 multiple, IUFD
Q0.00 Anencephaly-IUFD	Q03.9 Congenital hydrocephalus, unspecified + brain atrophy, premature
Q61.3 Polycystic kidney, unspecified	Q25 Transposition of the great arteries, TGA
Q01 Encephalocele	Q98.7 multiple
Q61.3 Polycystic kidney, unspecifiedHydrops fetalis not Rh	Q61.3 Polycystic kidney, unspecified + Intestinal obstruction
Q91.3 Edward syndrome	Q98.7 multiple, Premature
Q37 Ccenter palate with ccenter lip	Q98.7 multiple, Premature
Q87.8 Cystic Hygroma	Q61.3 Polycystic kidney, unspecified + mass in abdomen + asphyxia, dead
Q37 Ccenter palate with ccenter lip	Q74.2 congenital malformations of lower limbs
Q98.7 multiple+SB	Q74.2 congenital malformations of lower limbs + Abdominal wall
Q79.0 diaphragmatic hernia+Abdominal wall defect	Q37 Ccenter palate with ccenter lip + multiple
Q05 Spina bifida	Q05 Spina bifida
	Q61.9 Congenital single renal cyst

Previous BD are frequent in couples with a newborn with BD. For women with a M, or a newborn premature and still born, there was no significant history of previous BD. Recurrence of late miscarriages is high, about half of the cases of M. For women with a premature newborn or SB there is no significant history of previous M; these are instead increased in BD mothers (Table 4).

**Table 4:** Trend in the repetition in time of negative events during the reproductive life of the couple. Frequency of previous late micarriages, and previous BD in couples with a new born child. ^a^ % of couples with more than 1 BD child before the normal newborn child. These data represent a trend, are dependent from length and productivity of repoructive life of the families, and thus have to be taken not as absolute frequencies, nor as prevalence.

	Previous late miscarriages (%)	Previoud BD (%)
Normal	na	1.9
Birth Defect	18.0	20.0
Miscarriage	42.3	3.2
Premature birth	10.6	4.2
Stillborn	9.0	3.3
Normal in family with previous BD	10.9	0.2 ^a^

Sixteen couples among the 55 with a newborn with BD had previously a child with BD, or a collateral with BD. Eleven of them, with total of 168 children, had a previous child with BD themselves, with recurrence of BD in the same couple of 20%, and frequency among all the progeny of couples with a BD child of 17/168 of their children (10%). The BD that recur in these couples are of the same primary type in half of the cases (three cases of NT, one of CHD and one of CLP), but of a different type in the other couples (s-Table V).

Marriage of first cousins among the couples with recurrence of BD was 40%, slightly higher than that in the population, but not significant. Among these couples with recurring BD, one had also recurrence in the collateral family, for a different type of BD, and one for the same BD.

Seven couples with BD had nephews/nieces with BD. The collaterals of parents with BD, include 694 siblings, all healthy, and 1,423 children of their progeny. Among these last, 14 children had BD, with a frequency of 1%, not very different from that of incidence in the general population (Table 5 and s-Table V, left).

**Table 5:** Listing of couples with BD and PBD and/or BD in relatives. N, none; na. not available. Parent relation: 1-first cousins, 2-extended family, 3-unrelated.

BD in newborn	Parents relation	Previous BD/year	N sister children BD	N brother children BD	N sisters children BD	N brothers children BD
Q20	na	1child-Q20	0	0	0	0
Q02	1	1child Q20, 1y	0	0	0	0
Q41.2	3	1 child Q02; 1child Q04.3	0	0	0	0
Q03	3	1 child Q61.3	na	na	na	na
Q00.0	1	1 child Q20, CHD, IUGF; 1 child Q04.3	0	0	0	
Q03	3	3 children Q03/18,11,10 y	0	0	0	0
Q75.9	1	2 children Q04.9/3,1y	0	0	0	
Q75.9	2	1 child Q03	0	0	0	0
Q03.9	3	# Q03/18 to 10y	0	0	0	0
Q05	Na	N	1child Q04.3	0	0	0
Q39.0	3	N	0	0	0	1child Q03, 2 children dumbness
Q61.3	3	N	0	0	0	
Q87.0	1	N	0	0	0	Q37
Q75.9	1	N	0	0	1, Q01.9	0
Q37	1	Y, 2 children multiple /7,8 y	1 child Q04.3	0	0	1 child Q04.3
Q39.0	2	N	4 children Q00.0	0	0	1 child Q04.3

### 2.3. Residence and BD

**Figure 2:**
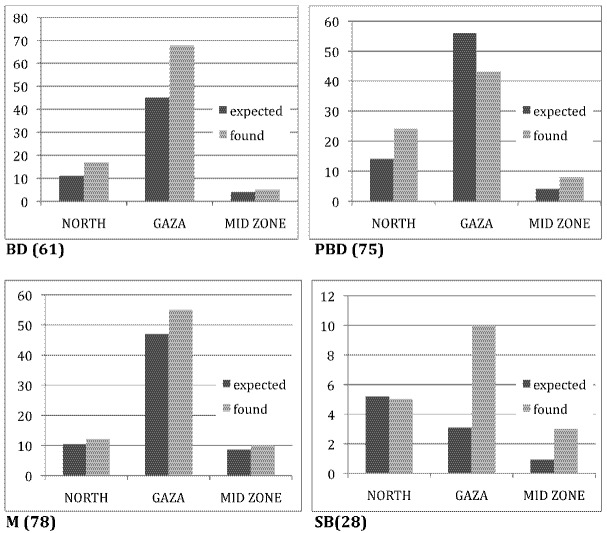
Distribution of the residence of the couples of newborn with Birth Defect (BD), and of late Miscarriages (M), Still Born (SB) and Previous Birth Defects (PBD).

The territory from which women come at Al Shifa maternity includes North, Gaza and Middle areas. The incidence with respect to territorial distribution of couples with negative reproductive events was balanced against the territorial distribution of all women admitted to delivery. The difference between expected prevalence per area and that found are illustrated in Figure 2. These are provisional indications that prevalence of BD, M and SB may be occurring preferentially in Gaza city, while the PBD in the North. It is needed to collect the data from more deliveries with BD in order to obtain significance of these differences.

The expected value is calculated on the basis of the distribution of residence of the couples with healthy newborns that reflects the basin of provenience of the people that utilize the Al Shifa Maternity. This is compared with the actual numbers of couples with a negative reproductive event coming from each of these areas. In bracket the number of cases for each event.

### 2.4. Environmental Risks and Correlation to BD Occurrence

Almost 3,000 delivering women were asked and responded to the question of if they had exposure to WP, and we could compare the frequency of exposure of couples with normal children with that of couples with BD. The exposure to WP in the 2008/2009 military attacks correlated significantly with the occurrence of BD (Table 6 and s-Table V, right, and s-Table VI).

**Table 6:** Exposure to WP and bombing *.

Exposure		None		only WP **		only Bombed			WP and Bombed
	Total	Number	Prevalence (95% CI)	Number	Prevalence (95% CI)	Number	Prevalence (95% CI)	Number	Prevalence (95% CI)
Normal	2,933	2,884	98.3 (97.9–98.8)	49	1.7 (1.2–2.1)	N/A		N/A	
BD	44	19	43.1 (28.5–57.8)	12	27.2 (14.1–40.4)	9	20.4 (8.5–32.4)	8	18.2 (6.8–29.6)
Overall	2977	2,903	97.5 (97.0–98.1)	61	2.0 (1.5–2.6)				

Reported exposure to WP during operation Cast lead was registered for 3 months for all the mothers in the delivery room. For families of BD children it was registered exposure to WP and to bombing during the five months. N/A not available * Incidence are expressed as percent of respective total and 95% Confidence Intervals ** The difference between exposure for parents of BD children *vs*. those of normal children is highly significant, *p* < 0.001.

We cannot say if the distribution in kinds of BD was affected in families exposed to bombing and/or WP, due to small numbers.

**Figure 3:**
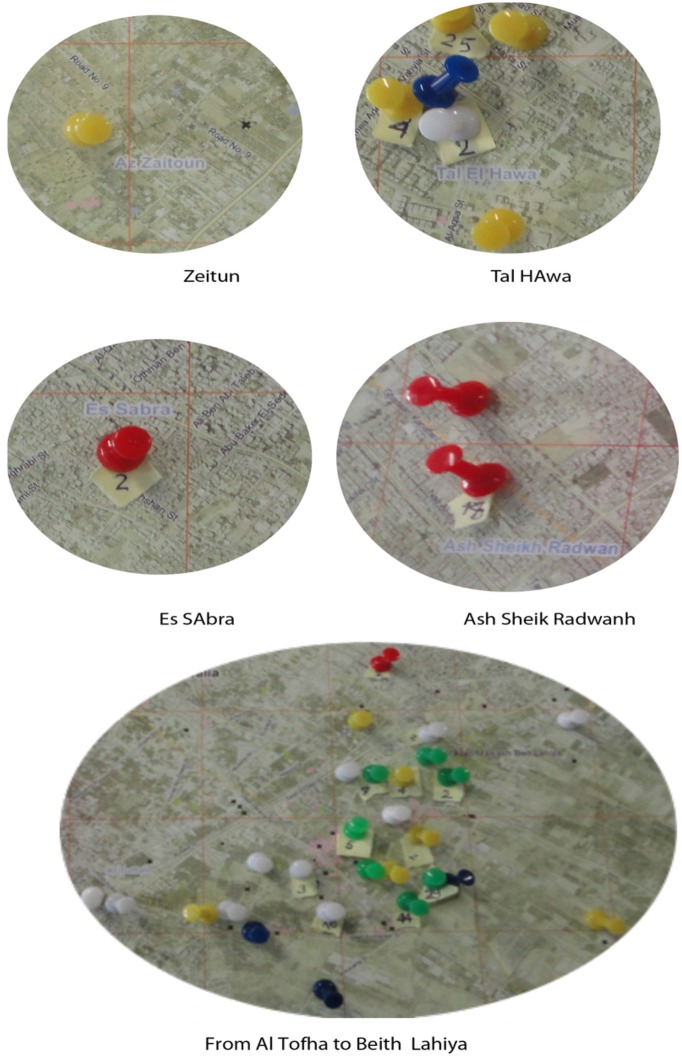
Mapping of the exposure to weaponry.

The subjective recollection of the exposure to WP and to shelling was confirmed for all but one of the couples confronting their recollection with information offered by the UN Mine Action Team from their data base and visually reported with a color code according to the OCHA GPS coordinates on the map of Gaza strip. In response to private and public call, the UN team documented WP ammunition, bombs or mines shells. Their records are thus a minimum estimate of what was delivered in attacks. Figure 3 illustrates examples of areas of residence of the families with BD children that declared exposure to WP or Bombs fro which correspondence was found in all but 1 case.

Locations where residents have declared exposure to WP and Bombing are illustrated as example of the method we used to confirm correspondence between declaration of the couples and facts on the ground. Maps from OCHA, squares of 1 km^2^, data base UNSCO, UN Mine Action Team, Gaza: red pins-MK bombs (Mk 82, 83, 84) and M117 bombs; white pins, White Phosphorus 120 mm and 155 mm ammunitions; green pins, tanks and 155 mm projectiles HE; yellow pins, shells of all kinds found in rubble clearing; blue pins, various projectiles. Enlargement of last panel is 0.5× with respect to the first four panels.

Among the 46 couples with BD child responding also to other questions on environmental exposure, 13 rescued their items from rubble of bombed places, nine rebuilt with rescued material, five were exposed to weapons or debris of bombing through work and in three couples one of the parents was injured during Cast lead. Also the couples with PBDs reported with high frequency exposure to WP or/and Bombings (s-Table V, right).

The main source of drinking water was that of the municipality pipelines for all the families and no occupational biases were observed.

## 3. Experimental Section

Our study was wholly conducted in Al Shifa Hospital, in Gaza City, covering 44.4% of deliveries in governmental hospitals in the Gaza strip [1], with 17,997 births in 2010 [2] and about 20% of all births of the Strip. A team of trainee doctors covered 18 h/24 and 6/7days for five months and registered 4,027 deliveries and collected the written assent of to the treatment of their data for research report. This is a procedure approved by the local Ministry of Health, the structure to which the Al Shifa Hospital belongs for studies where no reference is done to personal data of patients and these cannot be identified. In most cases both parents signed the form, in some cases the father was absent and only the mother signed also on his behalf. Some individuals accepted only verbally, for mothers this happened usually because stressed by the delivery (all data had to be collected immediately after child birth, due to the rapid dismissal from the hospital after delivery for lack of space), or this was because not everybody was familiar with writing and asked the doctor to proceed registering their verbal consent, which was registered in the original files. A summary of the information given and the request of permission are included in the questionnaire, shown in its whole in Table 1 (in full in S-Table I). The first section of the questionnaire was filled for all deliveries and a second one was reserved for deliveries of a BD child, premature (P) and still births (SB) and in case of late miscarriages (M) and in the case that during the interview emerged that the couple with a normal newborn had previously a child with birth defect (PBD). Termination of pregnancies is not practiced in Gaza and miscarriages of less than 16 weeks are not registered, as occurs also in other national registries of countries where abortions are not allowed by the law or beliefs.

Clinical diagnosis were from the doctors in charge of the delivery room, confirmed, if appropriate, by the doctors in the Intensive care unit (equipped with pulse oxy-meters and portable ultrasound machine). We have registered the major structural BD according to the Eurocat/ICD10 nomenclature [25,26] grouped according to the primary defect [27,28]. This choice is in line with most registers available. Multiple malformations, are classified independently only if there is no obvious way to identify the primary defect. We refer for comparative purposes to the information provided in the 2009 report from ICHBDSR [28] based on the same criteria we used, and avoid reference to the data from countries where there is heterogeneity in the modalities of collection of data. M are deliveries after 16 weeks and before 28 weeks. These were spontaneous emergency cases and not diagnosis or exams where done on the fetus. P are children born below 1.5 kg, and if there was a birth defect the child was registered by us also among BD. SB are born dead after 28 weeks.

Statistics. Prevalence data are expressed as percentage; incidence rate for normal children, BD and premature births are expressed as number per 1,000 registered live births (3,919) while incidence rate for late miscarriages and stillborn is calculating using as denominator total deliveries (4,027). Comparisons between each subgroup and normal were performed using Fisher’s test. Confidence intervals are given and a *P* value < 0.05 was considered significant.

## 4. Conclusions

The prevalence of BD in Gaza strip is 14/1,000. Within the limits due to differences in diagnostic levels, dimension of samples and methodologies, this is comparable to that of less industrialized countries and lower that that reported for more industrialized US (30/1,000) and Europe (23/1,000) (s-Table VII). Information is not available to compare incidence of M; that of SB is comparable with other countries and premature born in Gaza are less frequent than reported in the USA (the highest in the developed world).

Ours was a pilot study to first assess prevalence of BD. Our approach using a pre-specified questionnaire seems adequate, with a good feasibility as testified by the short time to train participating doctors and the ease in completing the questionnaires by the mothers. The main limitation is related to the present circumstances in Gaza that allow diagnosing BD at birth almost exclusively on clinical basis. As a consequence, the prevalence of total BD we report here is underestimated. Underestimate is suggested strongly for CHD which emerges as the most frequent BD in the records of 0–2 year old patients of pediatric hospitals [29].

There are noticeable differences from other countries in prevalence in Gaza for two kinds of BD: considerably higher prevalence of congenital PKD, usually due to homozygosis for mutations in a single gene [12] and lower prevalence of genital defects. For these last in Al Shifa no cases of undescended testis were registered, while this accounts for the highest numbers of the genital defects in many other countries. The high prevalence of PKD, among renal defects, reveals a polymorphism in the PKHD1 gene, common to other Arab counties [13].

Distribution of most of other major BD is not significantly different in our register that in other countries (Supplementary Table VII, compiled from the 2009 report by ICHBDSR) [28]. Although intermarriage in Gaza occurs with high frequency, marriage between first cousins is not associated to occurrence of BD or M. Provisionally, we could not establish any specific association with first cousin marriage, as one would expect for a recessive genetic defect, even for PKD, a fact that requires further study. It will be possible to evaluate better the relevance of intermarriage with reference of each specific kind of BD once a larger size of the sample of registered BD will be obtained by establishing widely the at birth registration procedure. Increase of sample number will also expose the level of polymorphism in the population for frequent recessive alleles, or will be supportive of the relevance of local environmental factors.

We also report recurrence of BD within a couple, and that in the numerous siblings of the parents which is not significantly higher than in the general population, and the increase in frequency of M in couples with a BD child or with history of M. Nonetheless, even for the BD recurring in a couple, only in 50% of cases these are of the same primitive embryonic origin. Thus, seemingly, these recurrences of BD in the couple are often due to disjoint events of mutation or of epigenetic changes in a favorable genetic background or to variable phenotypic expression. At this stage we cannot distinguish among these possibilities. While the recurrence of a PKD could be attributed to parents heterozygosis, the fact that NTD, known as multifactorial and environmentally influenced, recur in the couple with similar or higher frequency suggest that high pre-existing genetic polymorphisms is only one of the mechanisms at the basis of BD reiteration.

The increase in M in the couples with BD agrees with the concept that often M are spontaneous abortion caused by BD, although we cannot prove this causality in lack of diagnosis on the aborted fetuses.

Overall, collaterals of couples with BD have no higher rate of BD than the general population and not always recurrence of the same type of BD, and only in some cases there is ground to assume a familiar genetic origin.

Both limited instances of familiar expression and modalities of recurrence of different BDs are compatible with a role for environmental events in the promotion of the incidence of (some) BD.

We show a strong correlation of BD newborns and parent’s exposure to attacks with WP, and a high frequence of exposure in these couples to bombing, and/or consequent rubble cleaning, and we retraced data showing the accuracy of the recollection of exposures. It emerges the strong suggestion that exposure to war-derived elements enhances BD occurrence at a successive time. Before being conclusive on the specific elements in these weaponry that may cause such effects, analysis of contamination by war-related toxicants needs to be done.

There is provisional indication that the incidence of BD, M and SB may be occurring preferentially in Gaza city, while PBD in the North. To confirm these differences in BD’s distribution, we need to collect the data from more deliveries with BD and we need to extend implementation of our questionnaire to maternities covering all the areas of the Gaza strip.

We present for the first time for Gaza strip, limited to the major maternity hospital, prevalence of major birth defects, late miscarriages, prematurely and still born children, the rates of survival in the short term, the sex ratio and intermarriage frequency in the population.

We present the validity of a protocol for data collection which allows to understand the modalities and to investigate the nature of events that produce the BD.

Through the collection of regionally relevant environmental exposures, we established correlation of negative reproductive events with confirmed exposure to war, a fact relevant for the etiology of the birth diseases in Gaza.

Since no previous prevalence data are available for this population or this Hospital, we do not have the possibility to compare the present data of incidence with the situation previous to the war of the winter 2008–2009, and we do not know if the prevalence is higher now than previous to war.

The correlation between children with defect at births and war exposure of their parents points to this possibility and rises serious concerns, which should be further investigated. Other potential teratogen elements in the environment are now also under study.

In conclusion, we show that in a majority of cases the birth defects occur as novel events without collateral family history and recur in the same couple and not in their collaterals, with different or same malformation, regardless of the consanguineity of parents and in strong correlation with exposure to White phosphorus and other bombing events.

## Supplementary Files

Supplementary File 1: ZIP-Document (ZIP, 1082 KB)
