# Supplementary material for: Birth Defects in Gaza: Prevalence, Types, Familiarity and Correlation with Environmental Factors
Source: Int J Environ Res Public Health. 2012 May 7;9(5):1732–47. doi: 10.3390/ijerph9051732 (PMC3386584; doi:10.3390/ijerph9051732)
Supplement: Supplementary File 1: — ZIP-Document (ZIP, 1082 KB) [file ijerph-09-01732-s001.zip › Naim et al.2011supplementary Table II BD .xls.pdf]

Supplementary Table II

Birth Defects in children

												MOTHER SIBILINGS						FATHER SIBILINGS			
interviewer N	BD n	BD classification*	child sex	alive	child number	parents relationship	date of birth	previous late miscarriages	previous BD/ year	N sisters	N sister children	N sister child with BD	N brothers	N brothers children	N brother child with BD	N sisters	N sister children	N sisters child with BD	N brothers	N brothers children	N brothers children with BD
E110	1	Q20 CHD	M	Y	5	na	17-08-11	N	Y 1 child-Q20, CHD	4	13	0	6	2	0	5	4	0	3	3	0
E229	6	Q02 Microcephaly + IUGR	F	Y	8	1	03-09-11	N	Y- 1 child Q02, Microcephaly	4	17	0	4	8	0	5	18	0	5	22	0
E275	7, 10	Q75.9 Congenital malformation of skull and face bones, unspecified + distended abdomen, premature	na	N	6	1	07-09-11	Y	N	1	2	0	7	0	0	7	15	1, Q01.9 Encephalocele	8	11	0
E286	11	Q41.2 ABD and Small bowel volvulus + menconium cyst	M	Y	13	3	08-09-11	N	Y-1 child Q03, Hydrocephalus; Q04.3 other reducitonof brain	1	6	0	2	24	0	3	5	0	6	41	0
E289	11	Q87.0 CL,abnormal ear & mouth shape , deformity in toes	M	Y	2	1	10-09-11	N	N	7	2	0	5	0	0	15	30	0	13	15	2 childrens withQ37
E354	11	Q89.7 and vaginal anomalies , abdominal distension , hydrops fetalis	F	Y	na	na	15-09-11	N	N	na	na	na	na	na	na	na	na	na	na	na	na
E38	11	Q98.7 multiple	F	Y	na	na	08-08-11	N	N	na	na	na	na	na	na	na	na	na	na	na	na
E380	7, 11	Q79.5 Congenital malformation abdomen + lung hyperplasia+	M	Y	1	3	18-09-11	N	N	5	3	0	4	0	0	2	6	0	5	2	0
E44	5	Q74.9 Unspecified congenital malformation of limbs	F	Y	3	3	08-08-11	N	N	5	2	0	2	6	0	0	0	0	3	2	0
E443	12	Q60.6 Potter syndrome and renal agensis, premature	F	Y	1	2	24-09-11	N	N	6	4	0	3	0	0	7	11	0	4	0	0
E465	6	Q03 Hydrocephalus	M	Y	6	3	27-09-11	Y	Y- 1 child Q61.3 ,Polycystic kidney, unspecified	na	na	na	na	na	na	na	na	na	na	na	na
E489	6	Q01.2 Occipital encephalocele	F	Y	na	na	28-09-11	N	N	na	na	na	na	na	na	na	na	na	na	na	na
E492	12	Q60.6 Potter's syndrome + hypoplastic lungs	F	Y	na	na	29-09-11	N	N	na	na	na	na	na	na	na	na	na	na	na	na
E96	10, 12	Q75.9 Congenital malformation of skull and face bones, unspecified + ascite+IUFD	M	N	3	1	15-08-11	N	N	8	21	0	4	12	0	4	7	0	8	0	0
F129	12	Q61.3 Polycystic kidney, unspecified	F	Y	6	3	22-05-11	N	N	1	7	0	8	22	0	3	19	0	8	30	2 children, congenital dumness and dyspasia
F234	6	Q0.00 Anencephaly-IUFD	F	N	3	1	03-06-11	N	N	2	0	0	2	0	0	1	0	0	3	2	0
F297	12	Q61.3 Polycystic kidney, unspecified	M	Y	3	1	09-06-11	N	N	4	1	0	6	5	0	3	9	0	4	5	0
F303	6	Q01Encephalocele	F	Y	2	3	09-06-11	N	N	0	0	0	6	0	0	2	4	0	3	6	0
F428	12	Q61.3 Polycystic kidney, unspecified	M	Y	4	na	19-06-11	N	N	3	3	0	3	1	0	3	1	0	5	1	0
F457	12	Hydropsys fetalis not Rh	F	Y	3	3	20-06-11	N	N	3	7	0	7	4	0	6	9	0	5	7	0
F459	11	Q91.3 Edward syndrome	F	Y	3	2	20-06-11	Y	N	9	30	0	4	11	0	1	2	0	3	13	0
F491	2	Q37 Cleft palate with cleft lip	M	Y	4	1	22-06-11	Y	N	5	24	0	3	16	0	2	13	0	5	16	0
F506	12	Q87.8 Cystic Hygroma	M	Y	1	1	27-06-11	N	N	3	0	0	5	0	0	3	3	0	0	0	0
F77	2	Q37 Cleft palate with cleft lip	M	Y	8	1	15-05-11	N	N	2	5	0	2	8	0	8	15	0	5	11	0
H1045	11	Q98.7 multiple+SB	M	N	2	2	14-08-11	N	N	2	4	0	3	5	0	7	na	0	7	na	0
H107	7	Q79.0 diaphragmatic hernia + Abdominal wall defect	M	Y	4	1	09-05-11	N	N	5	0	0	5	2	0	4	8	0	5	2	0 ^
H1122	4	Q05 Spina bifida	M	Y	1	na	21-08-11	N	N	na	na	na	na	na	na	na	na	na	na	na	na
H144	6,11	Q0 Ancephaly + multiple malformation (Q74.2 defect in knee joint and ankle joint) + ascites IUFD	F	N	na	na	18-05-11	Y	N	5	22	0	5	28	0	1	8	0	1	6	0
H169	5	Q74.2 congenital malformations of lower limbs	F	Y	3	2	21-05-11	N	N	6	20	0	2	6	0	2	0	0	8	10	0
H170	7	Q39.0 Atresia of oesophagus without fistula	F	Y	5	2	22-05-11	Y	N	4	8	4 children with anencephaly, Q00.0	5	5	0	2§	7	0	2	7	1 child Q04.3 other brain reduction
H196	7	Q39.0 Atresia of oesophagus without fistula, premature	F	Y	3	3	24-05-11	N	N	10	3	0	3	0	0	8	20	0	9	30	1child Q03, Hydrocephalus
H247	6	Q00.0 Anencephaly	M	Y	5	1	28-05-11	N	Y-1 child Q20, CHD,IUGF, and 1 child Q04.3, brain reduction	6	14	0	5	8	0	3	12	0	7	50	0
H499	4	Q05 Spina bifida	M	Y	1	na	18-06-11	N	N	8	16	1child Q04.3, brain atrophy	5	16	0	4	10	0	2	2	0
H500	7	Q30.0 Choanal atresia	na	na	na	na	18-06-11	na	na	na	na	na	na	na	na	na	na	na	na	na	na
H628	6	Q03.hydrocephalus	F	Y	9	3	27-06-11	N	Y- 3 children with Hydrocephalus, Q03/18,11,10y	2	11	0	5	25	0	6	41	0	4	25	0
H672	10	Q75.9 Facial bones anomaly	F	Y	3	1	03-07-11	N	Y- 2 children Q04.9 Cerebral Palsy/ 3,1 y	8	0	0	2	0	0	3	1	0	4	1	0
H704	2.11	Q37 Cleft palate with cleft lip + multiple	F	Y	1	3	07-07-11	N	N	na	na	na	na	na	na	na	na	na	na	na	na
H759	1	Q25 Transposition of the great arteries	M	Y	na	1	05-07-11	na	na	na	na	na	na	na	na	na	na	na	na	na	na
H761	12	Q62 Hydronephrosis	M	Y	2	3	16-07-11	N	N	1	5	0	2	0	0	1	3	0	2	1	0
H762	10	Q75.9 Facial bones anomaly	F	Y	4	2	14-07-11	N	Y-1 child Q03, Hydrocephalus	7	25	0	4	10	0	6	16	0	5	7	0
H779	2	Q37 Cleft palate with cleft lip	M	Y	9	1	16-07-11	Y	Y, 2 children multiple anomalies/7, 8 y	2	10	1 child Q04.3 brain athrophy	5	20	0	2	0	0	5	10	1 child Q04.3 brain athrophy
H781	2	Q37 Cleft palate with cleft lip, premature	F	Y	na	na	18-07-11	na	na	na	na	na	na	na	na	na	na	na	na	na	na
H796	11	Q98.7 multiple, IUFD	M	N	1	1	19-07-11	N	N	2	0	0	1	0	0	1	0	0	1	0	0
H797	6	Q03.9 Congenital hydrocephalus, unspecified + brain atrophy, premature	F	Y	10	3	20-07-11	N	Y.3 childern brain athropy, hydrocephalus Q03/ 18 to 10y	2	9	0	2	7	0	3	20	0	4	20	0
H799	1	Q25 Transposition of the great arteries, TGA	F	Y	na	1	20-07-11	na	na	na	na	na	na	na	na	na	na	na	na		na
H867	11	Q98.7 multiple	M	N	3	1	28-07-11	N	N	na	na	na	na	na	na	na	na	na	na	na	na
H941	12, 11	Q61.3 Polycystic kidney, unspecified + Intestinal obstruction	F	Y	1	3	04-08-11	N	N	6	1	0	5	5	0	5	17	0	5	6	0
H945	11	Q98.7 multiple ,Premature	M	Y	na	na	na	na	na	na	na	na	na	na	na	na	na	na	na	na	na
H947	11	Q98.7 multiple ,Premature	F	Y	2	3	06-08-11	Y	N	7	19	0	8	18	0	6	35	0	7	30	0
K133	12,1	Q61.3 Polycystic kidney, unspecified + mass in abdomen + asphyxia, dead	M	N	2	3	17-05-11	N	N	1	3	0	4	0	0	3	2	0	2	4	0
K588	10	Q74.2 congenital malformations of lower limbs	M	Y	1	3	28-06-11	N	N	5	2	0	1	0	0	5	3	0	5	4	0
K652	10, 7	Q74.2 congenital malformations of lower limbs + Abdomina wall	F	N	8	3	08-07-11	Y	N	4	20	0	1	0	0	1	10	0	4	19	0
K704	2.11	Q37 Cleft palate with cleft lip + multiple	F	Y	1	3	16-07-11	N	N	3	1	0	2	0	0	4	2	0	3	2	0
M311	4	Q05 Spina bifida	M	Y	1	2	06-09-11	N	N	1	0	0	1	0	0	7	0	0	2	0	0
M55	12	Q61.9 Congenital single renal cyst	F	Y	1	3	10-08-11	N	N	2	0	0	5	0	0	2	0	0	4	0	0
TOTAL BD 55			27F/28M	45Y/9N/1 na	168	16-1,7.2, 19-3 13-na		9Y/41N/5na	10 families Y, 40N, 5na for total 17 children	172	340	6 children with BD	164	274	0	164	386	1 child	194	423	7 children

Legend

* according to the listing as in the questionnarie used for interviews

^ father sister recurrent aboertions and father cousin with multiple anomalies

§ father sister with recurrent late miscarriages
