# Supplementary material for: Birth Defects in Gaza: Prevalence, Types, Familiarity and Correlation with Environmental Factors
Source: Int J Environ Res Public Health. 2012 May 7;9(5):1732–47. doi: 10.3390/ijerph9051732 (PMC3386584; doi:10.3390/ijerph9051732)
Supplement: Supplementary File 1: — ZIP-Document (ZIP, 1082 KB) [file ijerph-09-01732-s001.zip › Naim et al2011 extended Table I-Questionnaire .pdf]

***Attention interviewers** (the italic section is to be filled by you):*

A short explanation must be offered to the participants in this study to orient them. This will also put the participant in a more relaxed state of mind so they can best respond to our questions.

Please remember to get contact information for parents.
File number:
ID number:
Questionnaire Identification Number __________
This questionnaire contains information about the Child, father (FA) and mother (MO):

Interview date: ________________________________

Hospital: Al Shifa

Section I: (filled by the interviewer from the clinical record):

Place of residence of the family 1- North 2- Gaza 3 - Mid-zone 4- Kanyounis 5- Rafah

Locality: 1- City 2- Village 3- Camp 4-___________

Street or area ____

Reference Telephone _____________________________________

Type of family: 1- nuclear family 2- Extended family

Relation between parents: 1- cousins 2- from the family 3- no relation

***Sex of the child**:* 1- Male 2- Female

Birth date of the child:__________________________________

*Birth at __________**weeks of gestation***
This child: 1- Single 2- Twin 3- Triplet 4- Other Specify

Health status of this Neonate: 1- Excellent 2- good 3- fair 4- bad

Neonate Birth Weight _________ in Grams

Birth order of the neonate _______

Neonate delivery: 1- normal delivery 2- caesarean

Type of the malformation: circle all the appropriate if present in the list below if present- write the diagnosis if is congenital disease not in the list below

the neonate has

1- Congenital Heart Defect,CHD 2- Cleft lip /Palate , CL/P 3-Down Syndrome 4-Spina Bifida 5-Limb Defects 6- Neural tube defect, NT 7-Abdominal wall defect 8- Hypospadias 9-osteogenesis inperfecta and osteopetrosis, 10- skeletal defect, other than limb and CL/P 11- multiple malformations 12- Others: Specify _________

Additional clinical data:

Are any interventions required (i.e. therapeutic, surgical, or others)? --------------------------------------

Objective exams done ---

Referral to other Hospital departments or to other Hospitals---

***Section II:* for parents**

Begin here the interview starting by telling the parent the following:

We are starting a program of birth registration in the Hospital. This will produce information on the health status of the children population more at large if you collaborate giving also some additional information by answering to the questions below.

All the information you provide will be treated in strictest confidence and is only for health personnel and never your names or references will be disclosed. The information will be used for scientific and clinical purposes only.

Do you agree to answer?

Signature for consensus of Mother: ____

Signature for consensus of Father: ____

---

1. Sex of the child: M [ ] F [ ]
2. Date of birth: _____________________________
3.

-MOTHER

-Name: _ _________________

-Age : __ ________________

-Occupation present : __________________ Past: _____________________

-FATHER

-Name: ________________

-Age : ____________________

4. -Occupation present : __ ________________ Past: _____________________

How many children do you have?

	1	2	3	4	5	6	7	8	9	10	11	12
**Sex**												
**Age**												
**Birth Defect: year/kind**												
**Still born: year/ cause**												
**Death after birth: year/ cause**												
**With cancer: diagnosis**												
**With chronic diseases: specify**												

Did you lose any child before birth (miscarriage)? If yes, please indicate

How many: ______________________________

How long ago: ____________________________

How advanced was the pregnancy: ____

Did the foetus had defects? ____________________________________

Were you exposed to WP ?

Was your house hit with WP even if in your absence?

Additional question in case of stillborn child and miscarriages before this last child:

Do you have family or neighbours unrelated to you that have children with similar health problems as your child?

Only for families with a child with congenital birth defect or previous children with congenital birth defects, please fill Sections II and IV

For newborn children with malformations at birth: **PLEASE TAKE AND ATTACH A PICTURE OF RELEVANT FEATURES OF THE CHILD IN THIS QUESTIONNARIE**

Notice: for these children nail and hair collection has to be done

Section III: Only for parents with children with birth defect

Since there are many children with birth deformities, we want to look into the possible causes. We would like to ask you some questions to help us understand this situation. We also ask your consensus to collect a small amount of hair from your child and its nails form hands and feet. This will be done without any damage or risk for the child. In addition, the materials will then used, if necessary, for research studies.

We need the consensus of both mother and father to collect these samples.

Signature for Consent of mother: ______________________________<br>Signature for Consent of father: ______________________________

We also need to be able to contact you later. Please give us contact information.

MO section	FA section
– Name:________________________________<br>– Age:__________________________________<br>– Occupation<br>– present:_______________________<br>– Past:_________________________<br><br>– Do you suffer from any diseases?<br>– As a child, and an adolescent, did you have any especial health problems that stand out in your mind? Which?<br>____<br>– How many brothers you have? _______________<br>– How many sisters you have? _________________<br>– Are your brothers in general health?<br>Yes [ ] or No [ ]<br>– How many children they have?<br>– Are all of them healthy? If not please tell what they have?<br>– Have any of these children a birth disease? Describe<br>____<br>– If so, does he live near your residence?<br>____<br>– Are your sisters in general health?<br>Yes [ ] or No [ ]<br>– How many children they have? _____________<br>– Are all of them healthy? If not please tell what they have?<br>____<br>– Have any of these children a birth disease? Describe<br>____<br>– If so, does he live near your residence?<br>____<br>– Have any of your sisters or brother's wife had miscarriages with birth diseases? If so,, please tell what disease? ______________________________	– Name:________________________________<br>– Age:__________________________________<br>– Occupation<br>– present:_______________________<br>– Past:_________________________<br><br>– Do you suffer from any diseases?<br>– As a child, and an adolescent, did you have any especial health problems that stand out in your mind? Which?<br>____<br>– How many brothers you have? _______________<br>– How many sisters you have? _________________<br>– Are your brothers in general health?<br>Yes [ ] or No [ ]<br>– How many children they have?<br>– Are all of them healthy? If not please tell what they have?<br>– Have any of these children a birth disease? Describe<br>____<br>– If so, does he live near your residence?<br>____<br>– Are your sisters in general health?<br>Yes [ ] or No [ ]<br>– How many children they have? _____________<br>– Are all of them healthy? If not please tell what they have?<br>____<br>– Have any of these children a birth disease? Describe<br>____<br>– If so, does he live near your residence?<br>____<br>– Have any of your sisters or brother's wife had miscarriages with birth diseases? If so,, please tell what disease? ______________________________
– During your pregnancy, did you take painkillers? How many and how often?<br>____<br>____<br>– During your pregnancy, did you take antidepressants? How many and how often?<br>____<br>____<br>– What do you think about your diet during pregnancy?<br>____<br>____	– Do you have other spouses?<br>Yes [ ] or No [ ]. If yes, please answer<br>– How old is your second spouse? ____________<br>– How many children do you have with your second spouse?<br>____<br>– How old are the children with your second spouse?<br>____<br>– Have any of your children with your second spouse died, was malformed or had cancer (please tell us doctor's diagnosis if you know)?<br>____

Section IV: Environment & Public health Related Questions

1. **Where do you live now?**
- Is it 1-an apartment,2-house, 3- temporary recover?
- Is it at ground level?
- How long have you lived here?
- In which cities, towns, or localities have you resided since 2005?

2. **Where do you get your drinking water from (local well, or water pipes network), in each of the residences?**

3. **Were any of your residences bombed? Yes [ ] or No [ ]**

4. **If you answer (3) yes, were you at your residence during or after the attack?**
   During [ ] or After [ ]

5. **Were any of your neighbours houses bombed? Yes [ ] or No [ ].**

6. **Was your house attacked with white phosphorus? Yes [ ] or No [ ].**

7. **Were you ever burned, wounded, or injured? Yes [ ] or No [ ].**

8. **If (7) yes,**
   - What kind of injury?
   - Which part of your body?

9. **Was any other member of your household?**
   - Burned **Yes [ ] or No [ ].**
   - wounded **Yes [ ] or No [ ].**
   - killed? **Yes [ ] or No [ ].**

10. **Did you take immediate care of them or of any other wounded or killed individual?**
    Yes [ ] or No [ ].

11. **Did you**
    - Clean up the rubble from bombed/burned house? Yes [ ] or No [ ],
    - recovered your things from the rubble? Yes [ ] or No [ ],
    - rebuild the house on the rubble? Yes [ ] or No [ ],

12. **Have you built new rooms/house with recovered materials?** Yes [ ] or No [ ],

13. **Have you found strange objects nearby your residence?** Yes [ ] or No [ ],
    Describe ____

14. **Have you or any of you family worked in the field of reused building materials?**
    Yes [ ] or No [ ], **If yes**
    - **who?**
    - **What is his/her age?**

15. **Do your children play in bomb craters, buildings, construction sites or collected materials salvaged from sites that have been bombed? Yes [ ] or No [ ]**

16. **Did you experience any health problems during/after the attacks in 2006 and 2008-09? If you sought medical help or if you remember it clearly, please indicate which was the problem.**
