# Supplementary material for: Birth Defects in Gaza: Prevalence, Types, Familiarity and Correlation with Environmental Factors
Source: Int J Environ Res Public Health. 2012 May 7;9(5):1732–47. doi: 10.3390/ijerph9051732 (PMC3386584; doi:10.3390/ijerph9051732)
Supplement: Supplementary File 1: — ZIP-Document (ZIP, 1082 KB) [file ijerph-09-01732-s001.zip › Naim er al 2011 supplementary Table IV- PBD .xls.pdf]

Supplementary Table IV-Families with newborn child and previous BD in their reproductive history

									MOTHER SIBILINGS						FATHER SIBILINGS			
interviewer N	newbornchild	newborn diagnosis	date of birth	parents relation	PBD	previous late M	N sisters	N sisters children	N sister children BD	N brothers	N brothers children	N brother children BD	N sisters	N sister children	N bsister children BD	N brothers	N brothers children	N brothers children BD
E148	F	N	21-08-11	3	Y-1 child O03, Hydrocephalus	Y	7	8	0	5	4	0		0	0	6	3	0
E101	F	N	16-08-11	1	Y 1 child-O20, CHD	N												
E110	M	O20 CHD	17-08-11	3	Y 1 child-O20, CHD	N	4	13	0	6	2	0	5	4	0	3	3	0
E158	F	N	22-08-11	3	Y 1 child-O21.0 , CHD , vsd	N	4	17	0	2	6	0	6	17	0	3	5	0
E244	M	N	05-09-11	1	Y 1child osteoporosis	N												
E257	M	N	06-09-11	3	Y 1 child-O21.0, CHD , vsd	N	6	17	0	3	2	0	7	11	0	5	3	0
E286	M	N	08-09-11	3	Y-1 child O03, Hydrocephalus	N	1	6	1	2	24	0	3	5	0	6	41	2
E313	F	N	12-09-11	1	Y1 child O74.2 congenital malformations of lower limbs	N	9	22	1	5	0	0	3	1	0	5	5	0
E320	F	N	13-09-11	3	Y 1 child-O21.0 , CHD , vsd	N	7	15	0	6	27	0	10	20	0	4	1	0
E332	M	N	14-09-11	3	Y 1 child O65.0 Congenital hip dislocation	N	2	18	2	5	34	0	3	18	0	5	15	2
E4	M	N	04-08-11	3	*Y1 child Lymphoma*	N												
E409	F	N	21-09-11	3	Y 1child O04.9 Cerebral Palsy	N	3	24	0	2	11	0	7	35	0	3	6	0
E447	F	N	25-09-11	3	Y 1child O90 Down syndrome	N	2	0	0	7	15	3	5	8	0	4	11	0
F105	M	N	19-05-11	3	Y 1 child O02 Cerebral atrophy	Y												
F137	F	N	22-05-11	2	Y-1 child O03, Hydrocephalus	N												
F156	F	N	25-05-11	1	Y 1child O0 Ancephaly	N												
F251	M	N	06-06-11	1	Y 1 child-O20, CHD	N	5	0	0	4	0	0	2	0	0	6	2	0
F423	M	N	18-06-11	1	Y 1child O04.9 Cerebral Palsy	N	2	0	0	5	4	0	3	16	1	4	9	0
F48	M	N	13-05-11	3	Y 1child E84 (ICD10) Cystic fibroids	N	6	18	0	4	0	0	13	40	0	21	100	0
F55	F	N	13-05-11	1	Y1child Q13.3 Congenital corneal opacity	Y												
F584	M	N	10-07-11	3	Y1 child Q61.3 Polycystic kidney, unspecified	N	5	16	0	5	14	0	5	19	0	3	0	0
F65	F	N	14-05-11	3	Y 1 child Q65.0 Congenital hip dislocation	N												
F68	M	N	14-05-11	3	Y 1 child Unknown	N												
H1044	M	N	15-08-11	2	*Y 1child Mental retardation*	N	2	4	2	0	0	0	4	20	0	3	2	0
H1049	M	N	15-08-11	3	Y 1child O79.5 Abdominal wall defect	N	6	30	0	2	0	0	5	20	0	3	0	0
H1064	F	N	16-08-11	1	Y 1child Q39.0 Atresia of oesophagus without fistula	N	2	2	0	3	0	0	5	5	0	2	2	0
H1116	F	N	19-08-11	1	Y 1 child Unknown	N	3	8	0	4	8	0	1	0	0	4	9	0
H1161	F	N	23-08-11	3	Y 1 child Unknown	N	2	1	0	3	1	0	2	6	1	4	18	0
H1172	F	N	24-08-11	1	Y 1 child Unknown	N	3	1	0	4	0	0	4	15	0	2	1	0
H1291	F	N	08-09-11	1	Y 1child Q98.7 multiple	Y	5	35	0	2	11	0	5	30	0	8	70	0
H1333		N	04-09-11	1	Y 1child Q61.3 Polycystic kidney, unspecified	Y	5	4	0	2	0	0	3	15	0	1	5	0
H141	M	N	18-05-11	1	Y1 child delivery(cerebral haemorrhage)	N												
H1414	F	N	24-09-11	3	Y-1 child O03, Hydrocephalus	N	9	35	0	3	7	1	3	19	0	6	13	0
H1470	M	N	01-10-11	3	Y 1child Q98.7 multiple	N	6	2	1	9	7	0	6	20	0	4	8	0
H1496	F	N	04-10-11	1	Y1 child Q61.3 Polycystic kidney, unspecified	N	5	1	0	7	5	0	7	15	0	4	6	0
H211	M	N	24-05-11	3	Y1 child Q61.3 Polycystic kidney, unspecified	N	1	3	0	3	2	0	4	10	0	6	15	0
H212	M	N	24-05-11	3	Y-1 child O03, Hydrocephalus	Y	5	40	0	8	12	0	2	16	0	6	20	0
H249	M	N	28-05-11	3	Y 1 child Unknown	Y	5	25	0	4	35	0	4	20	0	8	50	0
H276	M	N	30-05-11	3	Y 1 child-O20, CHD	Y	2	10	1	4	20	0	3	18	0	5	13	1
H277	F	N	30-05-11	3	Y 1 child Unknown	N	5	20	0	8	20	0	4	11	0	6	20	1
H279	F	N	30-05-11	1	Y 1 child Q65.0 Congenital hip dislocation	Y	1	2	0	3	0	0	4	5	0	8	12	1
H368.1	M	N	07-06-11	3	Y 1 child Unknown	N	5	20	0	4	10	1	5	27	0	4	30	1
H426.1	M	N	12-06-11	3	Y 1 child Unknown	N	4	10	0	3	3	0	7	40	0	4	50	0
H431	F	N	12-06-11	1	Y-1 child O03, Hydrocephalus	N	10	60	0	5	50	0	5	15	1	2	0	0
H449	F	N	14-06-11	1	Y 1child Q98.7 multiple	N	5	1	0	6	2	0	7	41	1	3	7	0
H479	M	N	15-06-11	1	Y 1 child Unknown	N	4	11	0	3	8	3	2	0	0	2	2	0
H535	F	N	20-06-11	3	Y-1 child O03, Hydrocephalus	N	10	6	1	2	1	0	3	25	0	4	20	0
H554	F	N	21-06-11	3	Y 1 child-O20, CHD	N	2	6	0	4	16	0	4	20	0	4	25	0
H597	M	N	23-06-11	1	Y 1child O04.9 Cerebral Palsy	N	0	0	0	6	0	0	6	0	0	3	3	0
H628	F	O03.hydrocephalus	27-06-11	3	Y-1 child O03, Hydrocephalus	N	2	11	0	5	25	0	6	41	0	4	25	0
H637	F	N	27-06-11	3	Y-1 child O03, Hydrocephalus	N	8	18	0	6	6	0	4	14	0	3	6	0
H672	F	Q75.9 Facial bones anomaly	03-07-11	1	Y 1child O04.9 Cerebral Palsy	N	8	0	0	2	0	0	3	1	0	4	1	0
H689	F	N	27-06-11	3	Y 1child Q79.0 Congenital diaphragmatic hernia	N	0	0	0	0	0	0	3	12	0	1	6	1
H849	F	N	26-07-11	2	Y 1 child-O20, CHD	N	8	17	0	6	9	1	4	9	0	5	0	0
H859	M	N	27-07-11	1	Y 1 child Q82.9 Congenital malformation of skin, unspecified	N	5	5	0	3	7	0	4	12	0	6	7	0
H939	M	N	03-08-11	1	Y 1 child Unknown	N	5	10	0	6	6	0	4	5	0	4	2	0
H949	F	N	06-08-11	1	Y 1 child-O20, CHD	N	5	21	2	3	8	0	3	8	0	1	6	0
H973	F	N	07-08-11	3	Y 1child O60 Renal agenesis	N	1	2	0	4	6	0	5	0	0	4	0	0
M165	F	N	20-08-11	1	Y 1 child Unknown	N												
M204	M	N	23-08-11	3	Y 1 child Unknown	N												
M239	F	N	25-08-11	1	Y 1 child Unknown	N												
M340	F	N	07-09-11	2	Y 1child Q98.7 multiple	N												
M350P	M	N	10-09-11	2	Y 1 child-O20, CHD	N												
M353P	M	N	10-09-11	3	Y 1 child Unknown	N												
M365	F	N	11-09-11	2	Y 1 child Unknown	N												
M286	M	N	04-09-11	3	Y 1 child Unknown	N												
M389	F	N	12-09-11	1	Y 1 child Unknown	N												
M392	M	N	13-09-11	2	Y O21.0 Dextro cardia +vsd	N												
M416	M	N	17-09-11	2	Y 1 child-O20, CHD	N												
M423	M	N	17-09-11	1	Y 1 child-O20, CHD	N												
M499P	M	N	04-10-11	1	Y 1 child Unknown	N												
M51	M	N	10-08-11	3	Y 1 child Unknown	N												
M56	F	N	10-08-11	3	Y 1 child Unknown	N												
E229	F	Q02 Microcephaly + IUGR	03-09-11	1	Y1child O04.3, 1 child O02	N	4	17	0	4	8	0	5	18	0	5	22	0
E465	M	Q03 Hydrocephalus	27-09-11	3	Y-1 child O61.3	Y												
H247	M	Q00.0 Anencephaly	28-05-11	1	Y-1 child O20	N	6	14	0	5	8	0	3	12	0	7	50	0
H762	F	Q75.9 Facial bones anomaly	14-07-11	2	Y-1 chil O03	N	7	25	0	4	10	0	6	16	0	5	7	0
H247	M	Q00.0 Anencephaly	28-05-11	1	Y-1 child O20, CHD,IUGF, 1 child O04.3	N	6	14	0	5	8	0	3	12	0	7	50	0
H628	F	O03.hydrocephalus	27-06-11	3	Y-3 children with Hydrocephalus, O03	N	2	11	0	5	25	0	6	41	0	4	25	0
H762	F	Q75.9 Facial bones anomaly	14-07-11	2	Y-1 child O03, Hydrocephalus	N	7	25	0	4	10	0	6	16	0	5	7	0
H779	M	Q37 Cleft palate with cleft lip	16-07-11	1	Y, 2 children multiple anomalies	Y	2	10	1 child O04.3 brain athrophy	5	20	0	2	0	0	5	10	1 child O04.3 brain athrophy
H797	F	Q03.9 Congenital hydrocephalus, unspecified + brain atrophy, premature	20-07-11	3	Y,3 childern brin athropy, hydrocephalus O03	N	2	9	0	2	7	0	3	20	0	4	20	0
Total 82	41M/43F	12 BD/72 Normal		32 1°- 10 2°-39-unr.		9Y/ 73N	248	720	12	232	524	9	247	844	4	258	849	10
Number	sex ratio	Bd versus normal (N)	date of birth	parents relation d- dergree	previous BD classification	previous M	N sisters	N sisters children	N sister children BD	N brothers	N brothers children	N brother children BD	N sisters	N sister children	N bsister children BD	N brothers	N brothers children	N brothers children BD
