# Supplementary material for: Birth Defects in Gaza: Prevalence, Types, Familiarity and Correlation with Environmental Factors
Source: Int J Environ Res Public Health. 2012 May 7;9(5):1732–47. doi: 10.3390/ijerph9051732 (PMC3386584; doi:10.3390/ijerph9051732)
Supplement: Supplementary File 1: — ZIP-Document (ZIP, 1082 KB) [file ijerph-09-01732-s001.zip › NAim et al 2011, suplementary Table V recurrence of BD+ Env .xls.pdf]

interviewer N	BD classification*		child sxe	child n.	parents	previous late M	previous BD/ year	N sisters	N sister children	N sister children BD	N brothers	N brothers children	N brother children BD	N sisters	N sister children	N sisters children BD	N brothers	N brothers children	N brothers children BD
**BD+PBD**																			
E110	Q20 CHD		**M**	**5**	**na**	**N**	**Y 1 child-Q20, CHD**	**4**	**13**	**0**	**6**	**2**	**0**	**5**	**4**	**0**	**3**	**3**	**0**
E229	Q02 Microcephaly + IUGR		**F**	**8**	**1**	**N**	**Y- 1 child Q02, Microcephaly**	**4**	**17**	**0**	**4**	**8**	**0**	**5**	**18**	**0**	**5**	**22**	**0**
E286	Q41.2 ABD and Small bowel volvulus + menconium cyst		**M**	**13**	**3**	**N**	**Y-1 child Q03, Hydrocephalus; Q04.3 other reducitonof brain**	**1**	**6**	**0**	**2**	**24**	**0**	**3**	**5**	**0**	**6**	**41**	**0**
E465	Q03 Hydrocephalus		**M**	**6**	**3**	**Y**	**Y- 1 child Q61.3 ,Polycystic kidney, unspecified**	**na**	**na**	**na**	**na**	**na**	**na**	**na**	**na**	**na**	**na**	**na**	**na**
H247	Q00.0 Anencephaly		**M**	**5**	**1**	**N**	**Y-1 child Q20, CHD,IUGF, and 1 child Q04.3, brain reduction**	**6**	**14**	**0**	**5**	**8**	**0**	**3**	**12**	**0**	**7**	**50**	**0**
H628	Q03.hydrocephalus		**F**	**9**	**3**	**N**	**Y- 3 children with Hydrocephalus, Q03/18,11,10y**	**2**	**11**	**0**	**5**	**25**	**0**	**6**	**41**	**0**	**4**	**25**	**0**
H672	Q75.9 Facial bones anomaly		**F**	**3**	**1**	**N**	**Y- 2 children Q04.9 Cerebral Palsy/ 3,1 y**	**8**	**0**	**0**	**2**	**0**	**0**	**3**	**1**	**0**	**4**	**1**	**0**
H762	Q75.9 Facial bones anomaly		**F**	**4**	**2**	**N**	**Y-1 child Q03, Hydrocephalus**	**7**	**25**	**0**	**4**	**10**	**0**	**6**	**16**	**0**	**5**	**7**	**0**
H797	Q03.9 Congenital hydrocephalus, unspecified + brain atrophy, premature		**F**	**10**	**3**	**N**	**Y,3 childern brain athropy, hydrocephalus Q03/ 18 to 10y**	**2**	**9**	**0**	**2**	**7**	**0**	**3**	**20**	**0**	**4**	**20**	**0**
								34	95	**1**	30	84	**0**	34	117	**0**	38	169	
**BD+ BD in KIN**														34	117	0	38		
H499	Q05 Spina bifida		**M**	**1**	**na**	**N**	**N**	**8**	**16**	**1child Q04.3, brain atrophy**	**5**	**16**	**0**	**4**	**10**	**0**	**2**	**2**	**0**
H196	Q39.0 Atresia of oesophagus without fistula, premature		**F**	**3**	**3**	**N**	**N**	**10**	**3**	**0**	**3**	**0**	**0**	**8**	**20**	**0**	**9**	**30**	**1child Q03, Hydrocephalus**
F129	Q61.3 Polycystic kidney, unspecified		**F**	**6**	**3**	**N**	**N**	**1**	**7**	**0**	**8**	**22**	**0**	**3**	**19**	**0**	**8**	**30**	**2 children, congenital dumness and dyspasia**
E289	Q87.0 CL,abnormal ear & mouth shape , deformity in toes		**M**	**2**	**1**	**N**	**N**	**7**	**2**	**0**	**5**	**0**	**0**	**15**	**30**	**0**	**13**	**15**	**2 childrens withQ37**
E275	Q75.9 Congenital malformation of skull and face bones, unspecified, + distended abdomen		**na**	**6**	**1**	**Y**	**N**	**1**	**2**	**0**	**7**	**0**	**0**	**7**	**15**	**1, Q01.9 Encephalocele**	**8**	**11**	**0**
								27	30	**1**	28	38	**0**	37	94	**1**	40	88	6
**BD+ PBD+BD in KIN**																			
H779	Q37 Cleft palate with cleft lip		**M**	**9**	**1**	**Y**	**Y, 2 children multiple anomalies/7, 8 y**	**2**	**10**	**1 child Q04.3 brain athrophy**	**5**	**20**	**0**	**2**	**0**	**0**	**5**	**10**	**1 child Q04.3 brain athrophy**
H170	Q39.0 Atresia of oesophagus without fistula		**F**	**5**	**2**	**Y**	**N**	**4**	**8**	**4 children with anencephaly, Q00.0**	**5**	**5**	**0**	**2**	**7**	**0**	**2**	**7**	**1 child Q04.3 other brain reduction**
								6	18	5	10	25	**0**	4	7	0	7	17	

total kin	house bombed	WP attacks	wounded/ injuried	rescue/clean rubble	materials recovery	work exposure	children exposure	symptoms
	N	N	N	N	N	N	N	N
	N	N	N	N	N	N	N	N
	Y	Y	N	Y	N	N	N	N
							N	N
	Y	Y	N	Y	N	N	N	N
	N	Y	N	N	N	N	Y	N
	N	N	N	N	N	N	N	N
	Y	Y	N	Y	N	Y	Y	N
	Y	Y	N	Y	Y	N	N	N
602	4Y/4N	5Y/3N	8N	4Y/4N	1Y/7N	1Y/7N	2Y/6N	8N
	N	Y	N	N	N	N	N	N
	N	N	N	Y	N	N	N	N
	Y	N	N	N	N	N	N	N
	Y	Y	N	Y	Y	Y	Y	N
	Y	N	N	Y	N	N	N	N
390	3Y/2N	2Y/3N	5N	3Y/2N	1Y/4N	1Y/4N	1Y/4N	5N
	N	Y	N	N	N	N		
	N	Y	N	N	N	N	N	N
	/2N	1Y/1N	2N	2N	2N	2N	1N	1N
