# Supplementary material for: Birth Defects in Gaza: Prevalence, Types, Familiarity and Correlation with Environmental Factors
Source: Int J Environ Res Public Health. 2012 May 7;9(5):1732–47. doi: 10.3390/ijerph9051732 (PMC3386584; doi:10.3390/ijerph9051732)
Supplement: Supplementary File 1: — ZIP-Document (ZIP, 1082 KB) [file ijerph-09-01732-s001.zip › Naim et al 2011, supplementary Table VI env BD.xls.pdf]

Supplementary TABLE VI

BD

interviewer N	BD classification*	residence present zone	locality	ground floor	drinking wate source	house bombed	WP attacks	wounded/ injuried	rescue/clean rubble	materials recovery	work exposure	children exposure	symptoms	child sex	child number	parents relationship	date of birth	previous late miscarriages	previous BD/ year
E110	Q20 CHD	GAZA	CITY	N	Y	N	N	N	N	N	N	N	N	M	5	na	17-08-11	N	Y 1 child-Q20, CHD
E229	Q02 Microcephaly + IUGR	GAZA	CITY	N	Y	N	N	N	N	N	N	N	N	F	8	1	03-09-11	N	Y- 1 child Q02, Microcephaly
E275	Q75.9 Congenital malformation of s	NORTH	CAMP	N	Y	Y	N	N	Y	N	N	N	N	na	6	1	07-09-11	Y	N
E286	Q41.2 and Small bowel volvulus + r	NORTH	VILLAGE	Y	Y	Y	Y	N	Y	N	N	N	N	M	13	3	08-09-11	N	Y-1 child Q03, Hydrocephalus; Q04.3 other reducitonof brain
E289	Q87.0 CL abnormal ear & mouth shape , deformity in toes	NORTH	VILLAGE	Y	Y	Y	Y	N	Y	Y	Y	Y	N	M	2	1	10-09-11	N	N
E354	Q89.7 and vaginal anomalies , abdominal distension , hydrops fetalis	GAZA	CITY											F	na	na	15-09-11	N	N
E38	Q98.7 multiple	GAZA	CITY	N	N	N	N	N	N	N	N	N	N	F	na	na	08-08-11	N	N
E380	Q79.5 Congenital malformation abdomen + lung hyperplasia+	MID ZONE	CAMP	Y	Y	N	N	N	N	N	N	Y	N	M	1	3	18-09-11	N	N
E44	Q74.9 Unspecified congenital malformation of limbs	GAZA	CITY	N	Y	N	N	N	N	N	N	N	N	F	3	3	08-08-11	N	N
E443	Q60.6 Potter syndrome and renal agensis, premature	GAZA	CITY	Y	Y	Y	N	N	N	N	N	N	N	F	1	2	24-09-11	N	N
E465	Q03 Hydrocephalus	GAZA	CITY									N	N	M	6	3	27-09-11	Y	Y- 1 child Q61.3 ,Polycystic kidney, unspecified
E489	Q01.2 Occipital encephalocele	MID ZONE	CAMP									N	N	F	na	na	28-09-11	N	N
E492	Q60.6 Potter's syndrome + hypoplastic lungs	GAZA	CITY											F	na	na	29-09-11	N	N
E96	Q75.9 Congenital malformation of skull and face bones, unspecified + ascite+IUFD	GAZA	CITY	N	Y	N	N	N	N	N	N	N	N	M	3	1	15-08-11	N	N
F129	Q61.3 Polycystic kidney, unspecified	MID ZONE	CAMP	Y	Y	Y	N	N	N	N	N	N	N	F	6	3	22-05-11	N	N
F234	Q0.00 Anencephaly-IUFD	GAZA	CITY	Y	Y	N	Y	N	N	N	N			F	3	1	03-06-11	N	N
F297	Q61.3 Polycystic kidney, unspecified	GAZA	CITY	N	Y	N	Y	N	N	N	N	N	N	M	3	1	09-06-11	N	N
F303	Q01Encephalocele	GAZA	CITY	N	Y	N	N	N	N	N	Y	N	N	F	2	3	09-06-11	N	N
F428	Q61.3 Polycystic kidney, unspecified	GAZA	CITY	N	Y	N	Y	N	N	N	N	N	N	M	4	na	19-06-11	N	N
F457	Hydropsys fetalis not Rh	NORTH	CAMP	Y	Y	N	N	N	N	N	N	N	N	F	3	3	20-06-11	N	N
F459	Q91.3 Edward syndrome	GAZA	CITY	Y	Y	Y	N	N	N	Y	N	N	N	F	3	2	20-06-11	Y	N
F491	Q37 Cleft palate with cleft lip	GAZA	CITY	Y	Y	N	N	N	N	N	N	N	N	M	4	1	22-06-11	Y	N
F506	Q87.8 Cystic Hygroma	MID ZONE	CAMP	Y	Y	N	N	N	N	N	N	N	N	M	1	1	27-06-11	N	N
F77	Q37 Cleft palate with cleft lip	NORTH	CAMP	Y	Y	N	Y	N	N	N	N	N	N	M	8	1	15-05-11	N	N
H1045	Q98.7 multiple+SB	MID ZONE	VILLAGE	N	Y	Y	Y	Y	Y	Y	Y	N	N	M	2	2	14-08-11	N	N
H107	Q79.0 diaphragmatic hernia + Abdominal wall defect	GAZA	CITY	N	Y	Y	Y	N	Y	Y	Y			M	4	1	09-05-11	N	N
H1122	Q05 Spina bifida	NORTH	VILLAGE	N	N	N	N	N	N	N	N	N	N	M	1	na	21-08-11	N	N
H144	Q0 Ancephaly + multiple malformation (Q74.2 defect in knee joint and ankle joint) + ascite	GAZA	CITY	N	Y	N	N	N	N	N	N	N	N	F	na	na	18-05-11	Y	N
H169	Q74.2 congenital malformations of lower limbs	GAZA	CITY	N	Y	N	N	N	N	N	N	N	N	F	3	2	21-05-11	N	N
H170	Q39.0 Atresia of oesophagus without fistula	GAZA	CITY	N	Y	N	Y	N	N	N	N	N	N	F	5	2	22-05-11	Y	N
H196	Q39.0 Atresia of oesophagus without fistula, premature	GAZA	CITY	N	Y	N	N	N	Y	N	N	N	N	F	3	3	24-05-11	N	N
H247	Q00.0 Anencephaly	GAZA	CITY	N	Y	Y	Y	N	Y	N	N	N	N	M	5	1	28-05-11	N	Y-1 child Q20, CHD,IUGF, and 1 child Q04.3, brain reduction
H499	Q05 Spina bifida	GAZA	CITY	Y	Y	N	Y	N	N	N	N	N	N	M	1	na	18-06-11	N	N
H500	Q30.0 Choanal atresia	NORTH	VILLAGE									N	N	na	na	na	18-06-11	na	na
H628	Q03.hydrocephalus	MID ZONE	CITY	Y	Y	N	Y	N	N	N	N	Y	N	F	9	3	27-06-11	N	Y- 3 children with Hydrocephalus, Q03/18,11,10y
H672	Q75.9 Facial bones anomaly	GAZA	CITY	Y	Y	N	N	N	N	N	N	N	N	F	3	1	03-07-11	N	Y- 2 children Q04.9 Cerebral Palsy/ 3,1 y
H704	Q37 Cleft palate with cleft lip + multiple	GAZA	CITY											F	1	3	07-07-11	N	N
H759	Q25 Transposition of the great arteries	GAZA	CITY									N	N	M	na	1	05-07-11	na	na
H761	Q62 Hydronephrosis	GAZA	CITY	N	Y	N	Y	N	N	N	N	N	Y	M	2	3	16-07-11	N	N
H762	Q75.9 Facial bones anomaly	NORTH	VILLAGE	Y	Y	N	Y	N	Y	Y	N	N	N	F	4	2	14-07-11	N	Y-1 child Q03, Hydrocephalus
H779	Q37 Cleft palate with cleft lip	NORTH	VILLAGE	N	Y	N	Y	N	N	N	N			M	9	1	16-07-11	Y	Y, 2 children multiple anomalies/7, 8 y
H781P	Q37 Cleft palate with cleft lip, premature	GAZA	CITY											F	na	na	18-07-11	na	na
H796	Q98.7 multiple, IUFD	GAZA	CITY	Y	Y	N	Y	N	Y	Y	N	N	N	M	1	1	19-07-11	N	N
H797p	Q03.9 Congenital hydrocephalus, unspecified + brain atrophy, premature	GAZA	CITY	Y	Y	Y	Y	N	Y	Y	N	N	N	F	10	3	20-07-11	N	Y,3 childern brain athropy, hydrocephalus Q03/ 18 to 10y
H799	Q25 Transposition of the great arteries, TGA	NORTH	VILLAGE									N	N	F	na	1	20-07-11	na	na
H867	Q98.7 multiple	NORTH	VILLAGE											M	3	1	28-07-11	N	N
H941	Q61.3 Polycystic kidney, unspecified + Intestinal obstruction	GAZA	CITY	Y	Y	N	N	N	N	N	N	N	N	F	1	3	04-08-11	N	N
H945	Q98.7 multiple ,Premature	MID ZONE	CAMP	N	N	N	N	N	N	N	N	N	Y	M	na	na	na	na	na
H947	Q98.7 multiple ,Premature	GAZA	CITY	Y	Y	Y	Y	Y	Y	Y	Y	N	Y	F	2	3	06-08-11	Y	N
K133	Q61.3 Polycystic kidney, unspecified + mass in abdomen + asphyxia, dead	GAZA	CITY	N	Y	N	N	Y	N	N	N	N	N	M	2	3	17-05-11	N	N
K588	Q74.2 congenital malformations of lower limbs	GAZA	CITY	Y	Y	N	N	N	N	N	N	N	N	M	1	3	28-06-11	N	N
K652	Q74.2 congenital malformations of lower limbs + Abdomina wall	GAZA	CAMP	Y	Y	Y	Y	N	Y	N	N			F	8	3	08-07-11	Y	N
K704	Q37 Cleft palate with cleft lip + multiple	NORTH	CAMP	Y	Y	Y	Y	N	Y	Y	N			F	1	3	16-07-11	N	N
M311	Q05 Spina bifida	NORTH	CAMP	N	Y	N	N	N	N	N	N			M	1	2	06-09-11	N	N
														F	1	3	10-08-11	N	N
						Y13/N31<br>ONLY BOMB 9	Y20/N24<br>ONLY WP 12												

respondants

residence question total 61

exposure questions total 44
