# Supplementary material for: Birth Defects in Gaza: Prevalence, Types, Familiarity and Correlation with Environmental Factors
Source: Int J Environ Res Public Health. 2012 May 7;9(5):1732–47. doi: 10.3390/ijerph9051732 (PMC3386584; doi:10.3390/ijerph9051732)
Supplement: Supplementary File 1: — ZIP-Document (ZIP, 1082 KB) [file ijerph-09-01732-s001.zip › Naim et al 2011Supplementary Table VII.pdf]

Supplementary Table VII

Incidence/1000 live births

	NT	CHD	CL&CLP	GI	Genital	Renal	Limb	ABD
**GAZA-al Shifa**	**3**	**2,3**	**1,5**	**0,5**	**0,25**	**2,3**	**1**	**0,7**
Australia	2,5	1,87	1,72	0,75	9,2	0,77	2,6	1,35
Canada-Alberta	2,3	1,34	2,2	0,48	4,9	0,8	2,9	0,96
Canada-Brit.Col	2,3	0,95	1,71	1,36	7,2	0,02	1,7	1,27
Chile	0,75	0,37	1	0,69	1,9	0,07	3.15	0,83
Cuba	2,3	0,55	0,67	0,7	1,27	0,43	0,37	1,27
Czech republic	1,6	1,8	1,9	0,95	3,1	0,98	0,8	1,2
Finland	2	2	2	0,9	0,35	0.98	0,8	1,2
France-Paris	2,5	1,68	1,6	0,69	1,8	1,1	1,3	1,1
Germay-Saxon	1,9	1,2	1,8	0,96	1,2	0.92	1,8	0,8
Hungary	1,7	1,55	1,3	0,52	4,9	0,7	1,3	0,5
Iran	5,4	0,85	1	3,9	1,5	0,25	3,25	0,85
Ireland	1,2	1,6	1,7	0,75	1	0,5	1,1	0,87
Israel	0,9	1.2	0,65	0,75	3,5	0,15	0,3	0,6
Italy-EmiliaRom.	1,1	1,4	1,25	0,56	1,6	1	0,9	0,5
Japan	1,7	1,5	2,6	1,9	0,3	0,6	0,9	1,2
Malta	2,3	2,8	3	3	0,46	0,35	1	1
Mexico	2,3	0,2	1,33	1,1	0,9	0,36	1,8	1
New Zelanda	0,7	1	1,4	0,5	8,1	0,8	3,3	0,6
Norway	2	1,6	1,9	0,9	1,5	0,8	0,7	1,3
Russia	1,3	0,5	1,2	0,6	3	0,6	0,5	0,9
S.America ECLAM	3,6	0,5	1,9	0,7	2,6	0,6	1,2	1,9
Ukraine	2,9	1	1,1	0,5	3,4	0,5	1	0,6
USA-Atlanta	2	1,5	1,2	0,77	1,1	0,5	0,65	1,1

Legend

The data here summarized illustrate incidence only for some BD, registered in 2007, and are taken from the ICHBDSR report 2009. Data include ToP with BD, when applied.
