# Supplementary material for: Birth Defects in Gaza: Prevalence, Types, Familiarity and Correlation with Environmental Factors
Source: Int J Environ Res Public Health. 2012 May 7;9(5):1732–47. doi: 10.3390/ijerph9051732 (PMC3386584; doi:10.3390/ijerph9051732)
Supplement: Supplementary File 1: — ZIP-Document (ZIP, 1082 KB) [file ijerph-09-01732-s001.zip › Naim et al.2001 supplementary Table III-M+P+SB .xls.pdf]

Supplemantary Table III

Miscarriages past 16weeks and before 28 weeks

interviewer file number	date of birth	child sex	alive at birth	parents relationship	previous miscarriages	previous BD	BD
E173	23-08-11	F	N	na	N	N	
E174	23-08-11	M	N	na	N	N	
E21	07-08-11	M	N	3	N	N	
E229	03-09-11	F	N	1	N	Y O04.3brain atrophy	
E237	04-09-11	F	N	3	N	N	
E275	07-09-11	na	N	1	Y	N	
E319	13-09-11	F	N	1	Y	N	
E380	18-09-11	M	N	3	N	N	
E393	20-09-11	F	N	3	N	N	
E401	20-09-11	F	N	1	Y	N	
E405	21-09-11	F	N	1	N	N	
E451	25-09-11	F	N	3	N	N	
E57	11-08-11	M	N	1	N	N	
E96	15-08-11	M	N	1	Y	N	
F214	31-05-11	M	N	na	na	N	
F234	03-06-11	F	N	1	N	N	
F260	06-06-11	M	N	1	Y	N	
F291	09-06-11	M	N	3	N	N	
F574	09-07-11	F	N	1	N	N	
F578	10-07-11	M	N	3	N	N	
F605	13-07-11	M	N	1	N	N	
F616	16-07-11	M	N	1	N	N	
F662	24-07-11	F	N	3	N	N	
H1001	10-08-11	M	N	1	Y	N	
H1025	13-08-11	M	Y	3	Y	N	
H1033	13-08-11	F	Y	1	Y	N	
H1045	14-08-11	M	N	2	N	N	
H1058	14-08-11	M	Y	3	Y	N	
H1066	16-08-11	F	Y	2	Y	N	
H1067	16-08-11	F	Y	3	Y	N	
H1095	17-08-11	F	N	3	N	N	
H1102	18-08-11	F	Y	1	Y	N	
H1120,1	19-08-11	M	N	3	N	N	
H1120,2	19-08-11	F	N	3	N	N	
H1160	23-08-11	M	N	3	Y	N	
H1170	24-08-11	M	Y	1	Y	N	
H1171	24-08-11	F	Y	3	Y	N	
H1179	24-08-11	M	Y	3	Y	N	
H1181	24-08-11	M	Y	3	Y	N	
H1191	25-08-11	F	Y	3	Y	N	
H121	16-05-11	M	Y	1	Y	N	
H1214	28-08-11	F	Y	1	Y	N	
H123	17-05-11	M	Y	2	Y	N	
H1280	07-09-11	M	N	3	N	N	
H1282	07-09-11	M	N	3	N	N	
H1283	07-09-11	M	N	na	N	N	
H1287	08-09-11	M	N	3	N	N	
H1288	08-09-11	F	N	3	Y	N	
H1322	12-09-11	na	N	1	N	N	
H1333	04-09-11	na	N	1	Y	YQ61.1Polycystic kidney	
H1340	14-09-11	F	N	3	Y	N	
H140-2	18-05-11	F	Y	2	Y	N	
H1418	25-09-11	F	N	1	N	N	
H147	12-06-11	F	N	3	Y	N	
H164	21-05-11	M	Y	3	Y	N	
H167	21-05-11	M	Y	3	3 x Y	N	
H191	23-05-11	F	Y	1	Y	N	
H194	23-05-11	F	Y	3	Y	N	
H239	27-05-11	F	Y	3	Y	N	
H241	27-05-11	F	Y	1	Y	N	
H260	28-05-11	F	Y	3	Y	N	
H268	30-05-11	M	Y	na	na	N	
H705	03-08-11	F	Y	1	Y	N	
H706,1	07-07-11	F	N	3	N	N	
H783	18-07-11	M	N	1	N	N	
H904	01-08-11	F	Y	3	Y	N	
H910	01-08-11	M	Y	1	Y	N	
H927	03-08-11	F	Y	1	Y	N	
H93	14-05-11	M	Y	1	Y	N	
H933	10-08-11	F	N	3	N	N	
K397	08-06-11		N	3	N	N	
K418	10-06-11	M	N	3	N	N	
K602	02-07-11	F	N	2	N	N	
K614	03-07-11	M	N	1	N	N	
K652	08-07-11	F	N	3	Y	Q74.2 lower limbs + Abdomina wall	
K662	10-07-11	F	N	3	N	N	
K738	22-07-11	M	N	1	N	N	
K739	22-07-11	M	N	1	N	N	
K762	30-07-11	M	N	1	N	N	
M116	15-08-11	M	N	3	N	N	
M160	20-08-11	M	N	3	N	N	
M213	23-08-11	na	N	3	N	N	
M247	27-08-11	F	N	3	N	N	
M251	27-08-11	F	N	3	N	N	
M283	04-09-11	M	N	1	N	N	
M318	06-09-11	M	N	3	N	N	
M328	06-09-11	M	N	2	N	N	
M345	08-09-11	M	N	3	N	N	
M346	08-09-11	M	N	3	N	N	
M406	14-09-11	M	N	3	N	N	
M445	19-09-11	M	N	3	N	N	
F459	20-06-11	F	Y	2	Y	N	Q91.3 Edward syndrome
F491	22-06-11	M	Y	1	Y	N	Q37 Cleft palate with cleft lip
H170	22-05-11	F	Y	2	Y	N	Q39.0 Atresia of oesophagus without fistula
TOTAL 94		48M/43F, 3na	31 Y, 63N	34 1°d, 8 2°-3° d, 47unrelated, 5 na	39Y, 53 N, 2na	3Y/91N	3Y/91N

PREMATURES past 28 weeks

interviewer file number	date of birth	child sex	alive at birth	parents relationship	previous miscarriages	previous BD	BD
E380	18-09-11	M	N	3	N	N	
E443	24-09-11	F	Y	2	N	N	
E456	26-09-11	F	Y	1	N	N	
E507	03-10-11	M	Y	3	N	N	
H677,1P*	03-07-11	F	Y	3	N	N	
H677,2P*	03-07-11	M	Y	3	N	N	
H904	01-08-11	F	Y	3	Y	N	
H1098,1P*	18-08-11	F	Y	1	N	N	
H1098,2P*	18-08-11	F	Y	1	N	N	
M2	06-08-11	M	Y	3	N	N	
M32	09-08-11	M	Y	3	N	N	
M79	13-08-11	M	Y	2	N	N	
M147	18-08-11	F	Y	3	N	N	
M150	18-08-11	M	Y	3	N	N	
M168	20-08-11	M	Y	3	N	N	
M235	25-08-11	M	Y	3	N	N	
M236	25-08-11	M	Y	2	N	N	
M279	04-09-11	F	Y	2	N	N	
M284	04-09-11	F	Y	2	N	N	
M285	04-09-11	F	Y	3	N	N	
M296	05-09-11	F	Y	3	N	N	
M300	05-09-11	F	Y	3	N	N	
M304	05-09-11	F	Y	2	N	N	
M317	06-09-11	F	Y	3	N	N	
M338	07-09-11	F	Y	3	N	N	
M350	10-09-11	M	Y	2	N	Y, Q20, CHD, 2y ago	
M353	10-09-11	M	Y	3	N	Y, BD not described, 15 y ago	
M371	12-09-11	F	Y	3	N	N	
M376	12-09-11	F	Y	2	N	N	
M380	12-09-11	F	Y	1	N	N	
M387,1*	12-09-11	F	Y	2	Y	N	
M387,2*	12-09-11	F	Y	2	Y	N	
M397	14-09-11	F	Y	3	N	N	
M420	17-09-11	M	Y	1	N	N	
M439	19-09-11	F	Y	3	N	N	
M440	19-09-11	M	Y	2	N	N	
M441	19-09-11	M	Y	3	N	N	
M448	20-09-11	F	Y	3	N	N	
M450	20-09-11	F	Y	3	N	N	
M453	21-09-11	F	Y	2	N	N	
M478	25-09-11	M	Y	3	N	N	
M481	26-09-11	F	Y	3	N	N	
M503	04-10-11	M	Y	3	N	N	

M504	04-10-11	M	Y	3	N	N	
H196	24-05-11	F	Y	3	N	N	Q39.0 Atresia of oesophagus
H947	06-08-11	F	Y	3	Y	N	Q98.7 multiple
E275	07-09-11	na	N	1	Y	N	Q75.9 skull and face bones
TOTAL 47 (3 couples of twins*)		19M/28F 1na	45Y/2N	6 1°d,12 2°-3°d, 29 unrelated	5Y/42N	2 Y/45 N	3Y/42N

N.B. registration of prematures was carired on only for 3 months

STILL BORN at term

interviewer file number	date of birth	child sex	parents relationship	previous miscarriages	previous BD	BD
E1	04-08-11	F	3	N	Y, not described	N
E111	17-08-11	F	3	N	N	N
E262	06-09-11	F	3	N	N	N
E34	07-08-11	F	1	N	N	N
E392	20-09-11	F	3	N	N	N
E62	11-08-11	F	1	N	N	N
F109	19-05-11	M	3	na	N	N
F121	20-05-11	M	3	na	N	N
F31	12-05-11	F	3	na	N	N
H103	14-05-11	M	3	Y	N	N
H1057	15-08-11	M	3	N	N	N
H1218	28-08-11	F	1	N	N	N
H125	17-05-11	F	2	na	N	N
H1289	08-09-11	M	1	N	N	N
H1329	14-09-11	F	3	N	N	N
H162	21-05-11	F	0	na	N	N
H189	22-05-11	F	1	na	N	N
H217	26-05-11	F	3	na	N	N
H275	30-05-11	M	3	Y, 4 times	N	N
H385	07-06-11	M	1	N	N	N
H648	30-06-11	M	3	N	N	N
H96	15-05-11	M	0	na	N	N
M13	06-08-11	M	1	N	N	N
M187	22-08-11	F	3	N	N	N
M190	22-08-11	M	1	N	N	N
M379	12-09-11	F	3	N	N	N
M380	12-09-11	F	1	N	N	N
M71	11-08-11	M	3	N	N	N
E96	15-08-11	M	3	N	N	Q75.9 skull and face bones + ascite+IUFD
H1045	14-08-11	M	2	N	N	Q98.7 multiple+SB
TOTAL 30		14M/16F	9 1°d, 2 2°and 3° d, 17 not related, 2 na	20N/2Y/8na	1Y/29N	2Y/28N
