# Supplementary figures and images for: Birth Defects in Gaza: Prevalence, Types, Familiarity and Correlation with Environmental Factors
Source: Int J Environ Res Public Health. 2012 May 7;9(5):1732–47. doi: 10.3390/ijerph9051732 (PMC3386584; doi:10.3390/ijerph9051732)

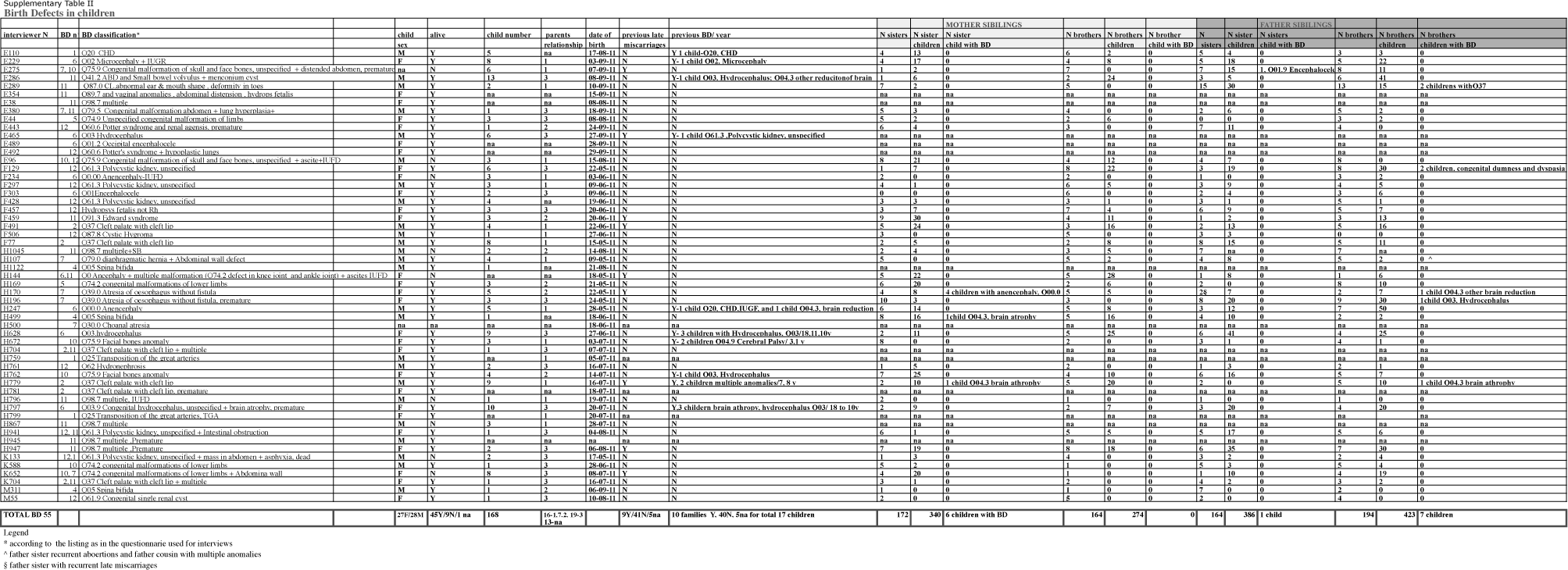

Supplement: Supplementary File 1: — ZIP-Document (ZIP, 1082 KB) [file ijerph-09-01732-s001.zip › Table 2 Suppl.tif]

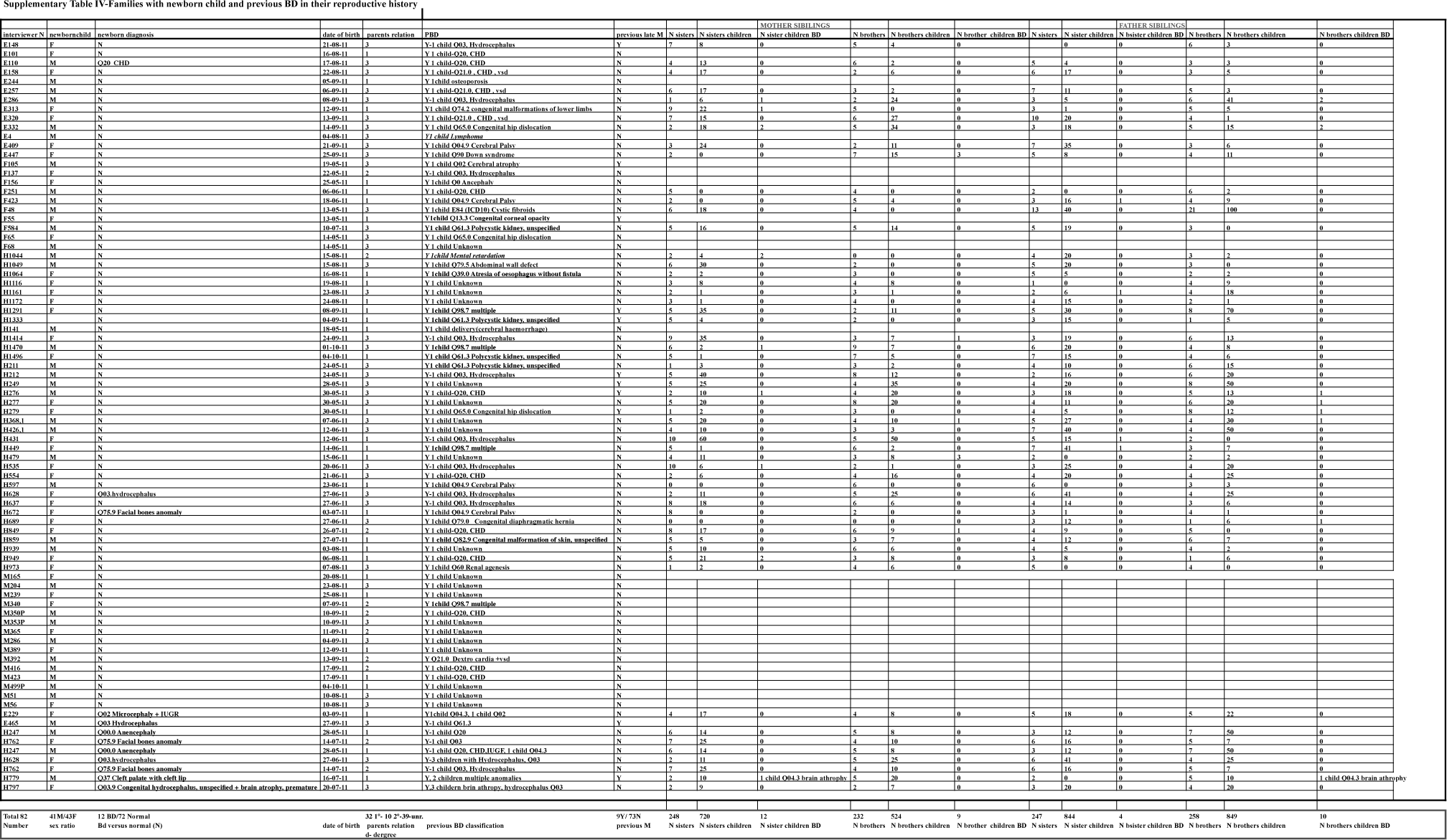

Supplement: Supplementary File 1: — ZIP-Document (ZIP, 1082 KB) [file ijerph-09-01732-s001.zip › Table 4 suppl.tif]

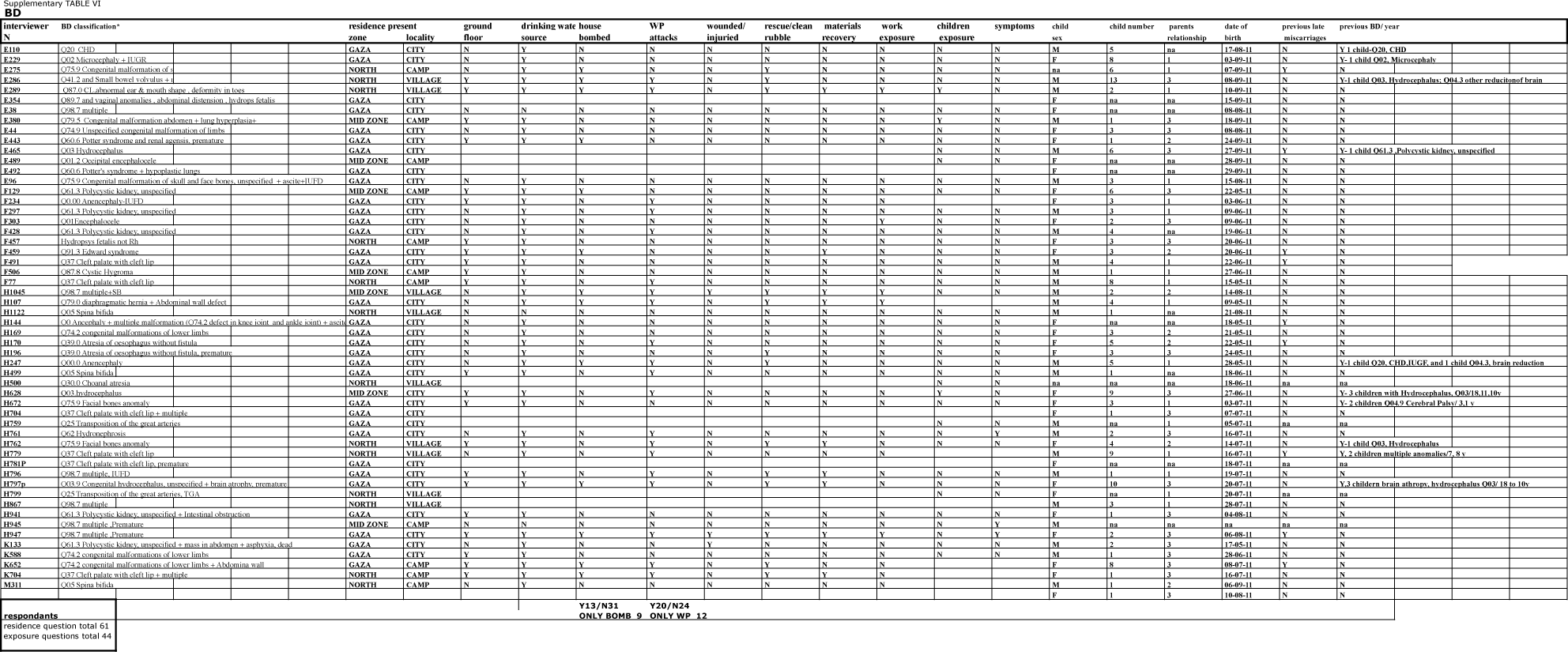

Supplement: Supplementary File 1: — ZIP-Document (ZIP, 1082 KB) [file ijerph-09-01732-s001.zip › Table 6 Suppl.tif]

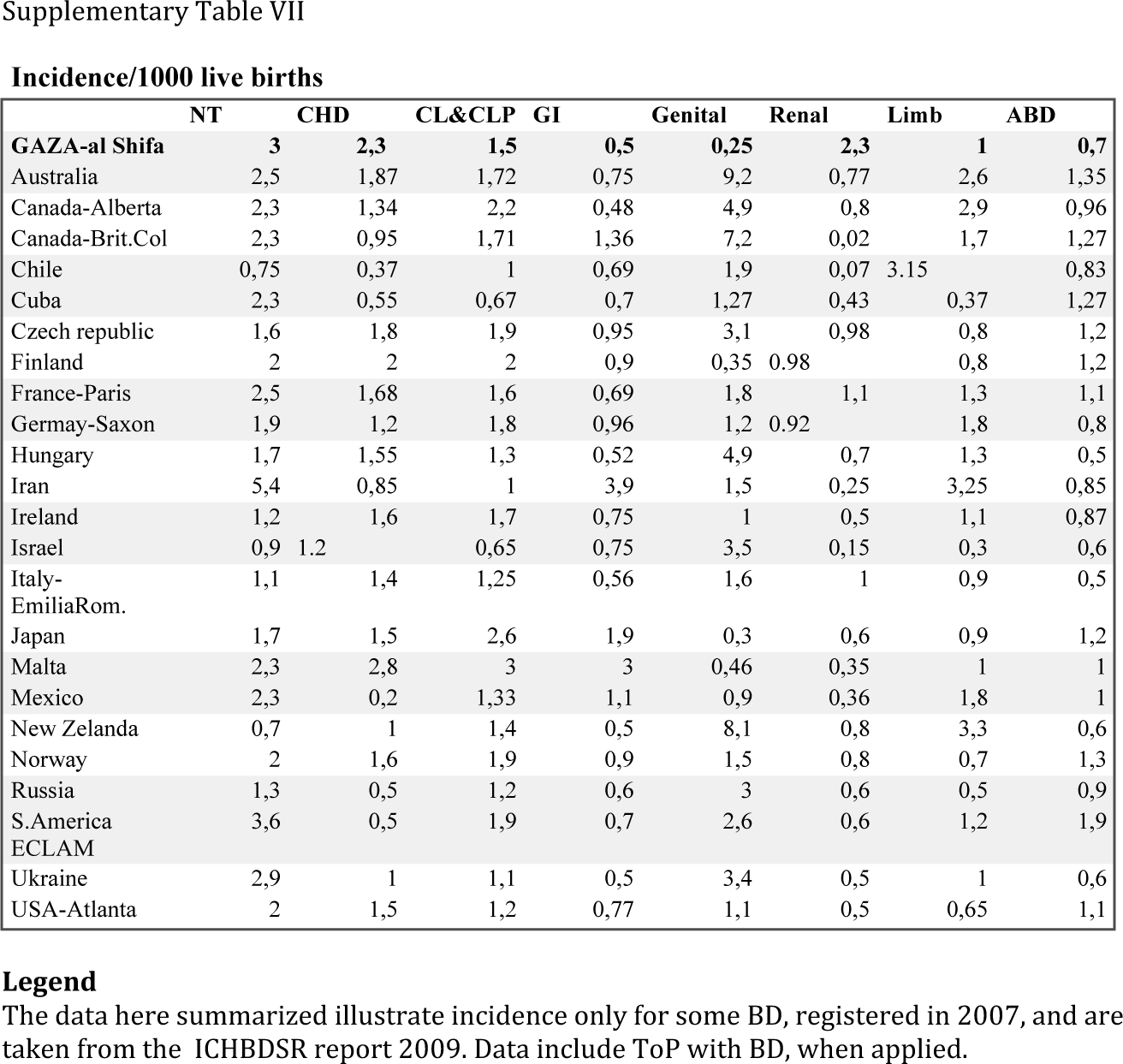

Supplement: Supplementary File 1: — ZIP-Document (ZIP, 1082 KB) [file ijerph-09-01732-s001.zip › Table 7 Suppl.tif]
